# Transcriptome-wide analysis of compression-induced microRNA expression alteration in breast cancer for mining therapeutic targets

**DOI:** 10.18632/oncotarget.8322

**Published:** 2016-03-24

**Authors:** Baek Gil Kim, Suki Kang, Hyun Ho Han, Joo Hyun Lee, Ji Eun Kim, Sung Hwan Lee, Nam Hoon Cho

**Affiliations:** ^1^ Department of Pathology, Yonsei University College of Medicine, Seoul, South Korea; ^2^ Brain Korea 21 Plus Project for Medical Science, Yonsei University College of Medicine, Seoul, South Korea; ^3^ Severance Biomedical Science Institute (SBSI), Yonsei University College of Medicine, Seoul, South Korea; ^4^ Department of Surgery, Yonsei University College of Medicine, Seoul, South Korea; ^5^ Global 5-5-10 System Biology, Yonsei University, Seoul, South Korea

**Keywords:** compression, microRNA, transcriptome, breast cancer, incurable cancer therapy

## Abstract

Tumor growth–generated mechanical compression may increase or decrease expression of microRNAs, leading to tumor progression. However, little is known about whether mechanical compression induces aberrant expression of microRNAs in cancer and stromal cells. To investigate the relationship between compression and microRNA expression, microRNA array analysis was performed with breast cancer cell lines and cancer-associated fibroblasts (CAFs) exposed to different compressive conditions. In our study, mechanical compression induced alteration of microRNA expression level in breast cancer cells and CAFs. The alteration was greater in the breast cancer cells than CAFs. Mechanical compression mainly induced upregulation of microRNAs rather than downregulation. In a parallel mRNA array analysis, more than 25% of downregulated target genes were functionally involved in tumor suppression (apoptosis, cell adhesion, and cell cycle arrest), whereas generally less than 15% were associated with tumor progression (epithelial-mesenchymal transition, migration, invasion, and angiogenesis). Of all cells examined, MDA-MB-231 cells showed the largest number of compression-upregulated microRNAs. miR-4769-5p and miR-4446-3p were upregulated by compression in both MDA-MB-231 cells and CAFs. Our results suggest that mechanical compression induces changes in microRNA expression level, which contribute to tumor progression. In addition, miR-4769-5p and miR-4446-3p may be potential therapeutic targets for incurable cancers, such as triple negative breast cancer, in that this would reduce or prevent downregulation of tumor-suppressing genes in both the tumor and its microenvironment simultaneously.

## INTRODUCTION

Tumor progression is associated with microRNA expression and signal transduction by tissue mechanics, or mechanotransduction [1]. However, little is known about the relationship between microRNA expression and mechanotransduction in tumor progression.

Tumor growth may induce aberrant expression of microRNA that leads to tumor progression via mechanotransduction. Mechanical stress, such as compression, tension, and interstitial fluid pressure, are expected to be increased by tumor growth, and thereby participate in tumor progression [2]. Indeed, increased compressive force was measured in the interior and periphery of tumors [3]. Compression-induced microRNA expression was previously reported in nontumor cells. In human periodontal ligament cells, expression of microRNA (miR)-29 is altered by compression [4]. miR-222 expression is upregulated by compression in articular cartilage [5], and mechanical compression induces upregulation of miR-146a in chondrocytes [6]. In breast cancer, miR-18a is mechanically upregulated by increased tissue stiffness [7], the major mechanical stress component of which is tension [8]. Whereas this finding does not constitute direct evidence to support compression-induced microRNA expression, it does support the possibility that tumor growth regulates microRNA expression level via compression.

Compromising compression-altered microRNAs may be a good option for cancer therapy. Aberrant expression of microRNAs is associated with tumor progression. Upregulation of miR-224 enhances tumor invasion and growth in non-small cell lung cancer by targeting SMAD4 and TNFα-induced protein 1 [9]. Upregulated miR-21 induces growth of hepatocellular cancer by modulating PTEN expression [10]. In breast cancer, miR-107 expression increases tumorigenesis and metastasis via inhibition of let-7 [11]. As a target for cancer therapy, the effect of microRNA modulation on tumor suppression has been validated in mouse models [12]. Uncontrolled proliferation is a fundamental characteristic of cancer cells [13]. Compression is likely one of the general stimuli leading to tumor progression [14]. Therefore, targeting compression-altered microRNAs may be useful for the development of a therapy that is generally applicable to various cancer phenotypes. Recently, personalized therapy has been a focus of cancer research [15–17]. This approach is expected to be highly effective in removing cancer cells, with reduced side effects. However, high cost and longer duration of treatment are thought to be drawbacks of personalized therapy. In this regard, cancer therapy targeting microRNAs commonly altered by compression may be a good alternative approach. To develop such a generally applicable cancer therapy, different responses of cancer cells to compression must be examined because tumor heterogeneity is one of the main causes of drug resistance [18–20]. In addition, the responses should be further investigated under different compressive states, which reflect the variation in compression during tumor growth.

In this study, we present microRNA transcriptome-wide analyses of compression-induced alterations in microRNA expression level in breast cancer cell lines [MCF-7(luminal A: ER+, PR+, HER2), BT-474(luminal B: ER+, PR+, Her2+), SK-BR-3(Her2: ER-, PR-, Her2+), MDA-MB-231(triple negative or Claudin-low: ER-, PR-, Her2-)] [21, 22] and cancer-associated fibroblasts (CAFs), a representative component of the tumor microenvironment, compressed at different relative compression units (RCUs). One RCU equals 5.8 mmHg (∼0.773 kPa), which is the approximate compression value of a native tumor microenvironment [23]. To investigate whether compression-induced microRNA expression contributes to tumor progression, the target genes of microRNAs identified in the parallel mRNA array analysis were further evaluated by classifying as tumor suppression-associated genes (TSAGs) and tumor promotion-associated genes (TPAGs).

## RESULTS

### Compression-induced alteration of microRNA expression level in breast cancer

**Figure 1:**
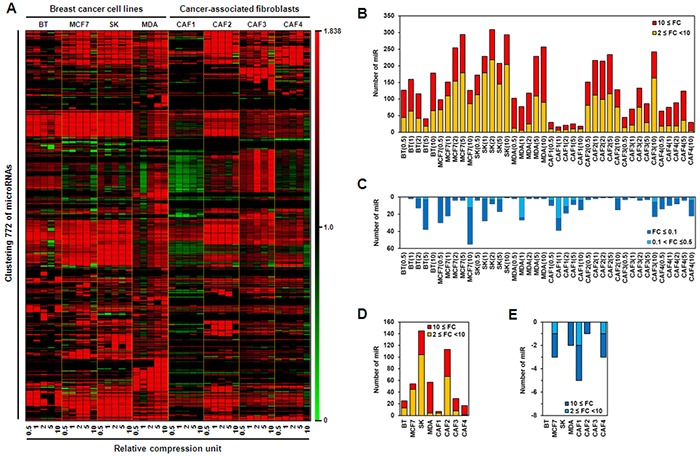
Compression-induced changes in microRNA expression level in breast cancer **A.** Pattern analysis of compression-induced microRNA expression level in breast cancer cells and cancer-associated fibroblasts (CAFs). Numerical comparison of **B.** compression-upregulated microRNAs, **C.** compression-downregulated microRNA, **D.** common compression-upregulated microRNAs at different compression conditions, and **E.** common compression-downregulated microRNAs at different compression conditions. For compression, 0 (control), 0.5, 1, 2, 5, or 10 RCUs were applied to cells for 24 h. BT, BT-474 cells; SK, SK-BR-3 cells; MDA, MDA-MB-231 cells.

Mechanical stress induces microRNA expression, leading to changes in cell behavior [24, 25]. Because tumor growth generates mechanical compression that acts on cancer cells and adjacent stromal cells, microRNA expression profiles in breast cancer cells and CAFs may be altered by compression. To confirm whether compression modulates microRNA expression, with changes dependent on the degree of compression, microRNA expression was examined by microRNA array in four types of breast cancer cell lines and four cultures of CAFs isolated from individual invasive ductal carcinoma (IDC) patients. As shown in Figure 1A, microRNA expression was up- or downregulated by compression in breast cancer cell lines and CAFs. Compression-upregulated microRNAs were generally more abundant in breast cancer cell lines than in CAFs, particularly those upregulated by 2- to 10-fold. MicroRNAs upregulated by compression by greater than 10-fold were similar among breast cancer cell lines and CAFs, with the exception of CAF1. CAF1 showed the lowest number of compression-upregulated microRNAs, whereas CAF2 showed the highest among CAFs (Figure 1B). Compression-downregulated microRNAs were much smaller in number than compression-upregulated microRNAs (Figure 1C). Compression-induced alterations in microRNA expression level were different at each RCU, and were not proportional to the degree of compression. Thus, the microRNAs commonly up- or down-regulated by compression were analyzed to compare cell susceptibility to compression and to develop diagnostic markers or therapeutic targets for cancer therapy. The largest number of microRNAs commonly upregulated 2- to 10-fold by compression was observed in SK-BR-3 cells, with the second largest number seen in CAF2. By contrast, the largest number of microRNAs commonly upregulated by greater than 10-fold was observed in MDA-MB-231 cells (Figure 1D). Less than 10 commonly downregulated microRNAs were seen in MCF7, MDA-MB-231, CAF1, CAF2, and CAF4 cells, and none were observed in BT-474, SK-BR-3, and CAF3 cells (Figure 1E).

### Analysis of cell-type–specific fold change in compression-upregulated microRNAs

**Figure 2:**
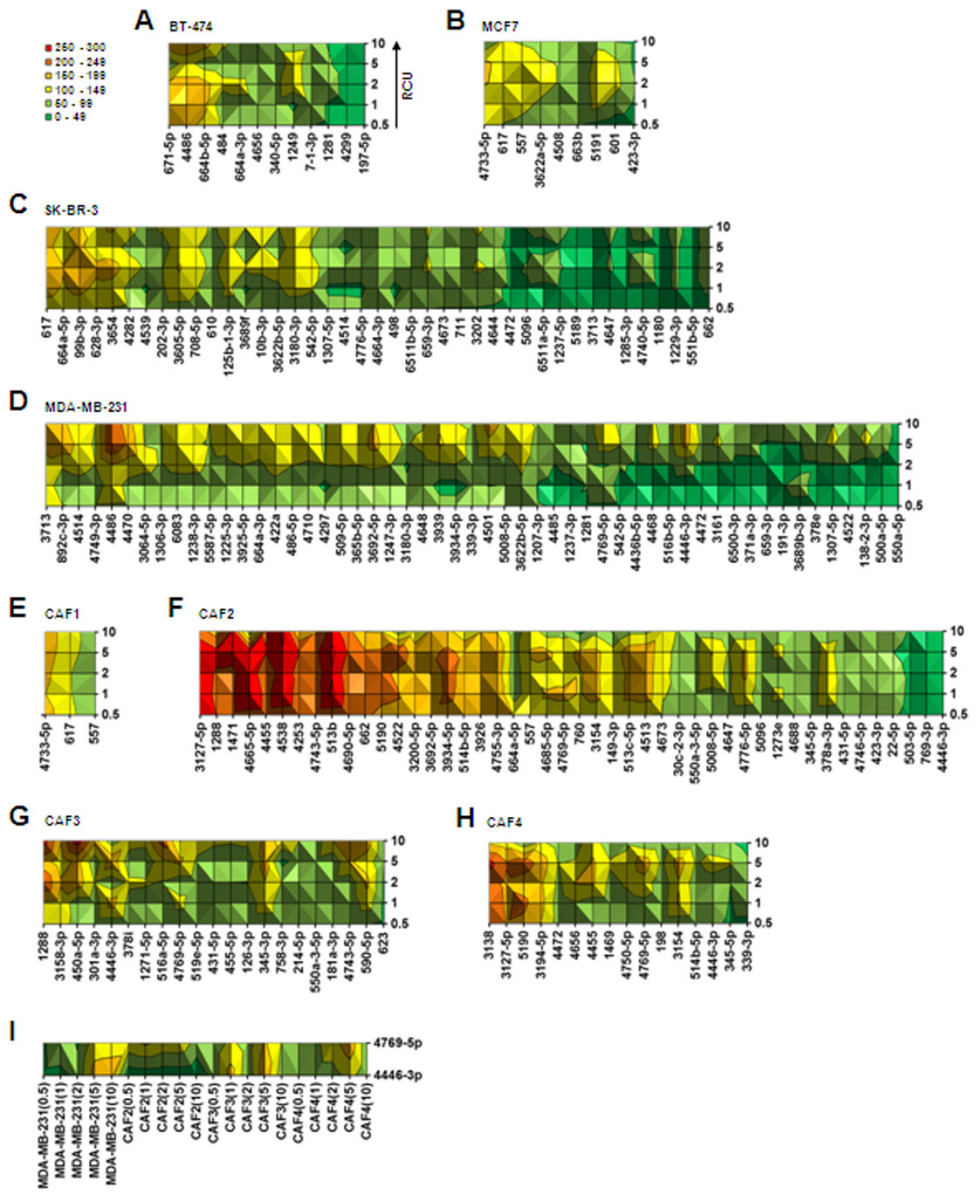
Analysis of cell-type–specific fold change in compression-upregulated microRNAs Surface graph analysis of compression-upregulated microRNAs in **A.** BT-474, **B.** MCF7, **C.** SK-BR-3, **D.** MDA-MB-231, **E.** CAF1, **F.** CAF2, **G.** CAF3, and **H.** CAF4. **I.** MicroRNAs commonly upregulated by compression in MDA-MB-231 cells and CAFs. The microRNAs showing greater than 10-fold upregulation were used for fold change analysis with six ranges: 0–49, 50–99, 100–149, 150–199, 200–249, and 250–300. For the analysis of commonly upregulated microRNAs in MDA-MB-231 and CAFs, average fold change values at all RCUs were used.

MicroRNA expression level was regulated by compression in breast cancer cell lines and CAFs (Figure 1A). Next, to understand how the degree of compression affects the level of microRNA expression, fold changes in expression of the microRNAs commonly upregulated by compression by greater than 10-fold were compared within six ranges of fold change: 0–49, 50–99, 100–149, 150–199, 200–249, and 250–300. Fold change values of compression-upregulated microRNAs are shown in Supplementary Table S1. Commonly downregulated microRNAs were not compared because they were insufficient in number (Figure 1E). In BT-474 cells, 12 microRNAs were upregulated by compression. MiR-671-5p, miR-4486, and miR-664b-5p showed the greatest fold increase, ranging from 100–199 (Figure 2A). Nine microRNAs were seen in MCF7 cells. MiR-4733-5p, miR-617, and miR-577 showed the greatest fold change, ranging from 100–149 (Figure 2B). In SK-BR-3 cells, 41 microRNAs were upregulated by greater than 10-fold by compression. MiR-617, miR-644a-5p, miR-99b-3p, miR-628, miR-3p, and miR-3654 showed the greatest fold change, ranging from 100–199 (Figure 2C). MDA-MB-231 cells showed the largest number (53) of compression-upregulated microRNAs among breast cancer cell lines. Interestingly, the fold change in microRNA expression level was generally higher at RCUs of 2, 5, and 10 than at 0.5 and 1 in MDA-MB-231 cells, unlike other breast cancer cell lines, which showed similar fold change at all RCUs. MiR-4486 showed the highest level of upregulation, ranging from 200–249 at RCUs of 5 and 10 (Figure 2D). In CAF1 cells, only three microRNAs were upregulated. MiR-4733-5p showed the largest fold increase, ranging from 150–199 (Figure 2E). CAF2 cells had the largest number of compression-upregulated microRNAs among CAFs (46) and the greatest fold increase among all cells examined. Fold change in expression levels of MiR-3127-5p, miR-1288, miR-1471, miR-4665-5p, miR-4538, and miR-513b in CAF2 ranged from 250–300 (Figure 2F). In CAF3 cells, 21 microRNAs were upregulated by compression. MiR-1288, miR-450a-5p, and miR-4446-3p showed the greatest fold increase (Figure 2G). In CAF4 cells, 16 microRNAs were upregulated by compression. MiR-3138, miR-3127-5p, and miR-5190 showed the greatest fold increase, ranging from 200–249 (Figure 2H). Simultaneously targeting cancer cells and the tumor microenvironment may be a good therapeutic strategy for currently incurable cancers, such as triple negative breast cancer: a heterogeneous group of breast cancer difficult to be treated by pre-existing chemotherapies for lacking estrogen receptor (ER), progesterone receptor (PR), and Her2/neu [26–29]. MiR-4769-5p and miR-4446-3p were commonly upregulated by compression in the triple negative breast cancer cell line MDA-MB-231, as well as in CAF2, CAF3, and CAF4 cells (Figure 2I).

### Analysis of fold change in expression levels of the putative target genes of compression-upregulated microRNAs

**Figure 3:**
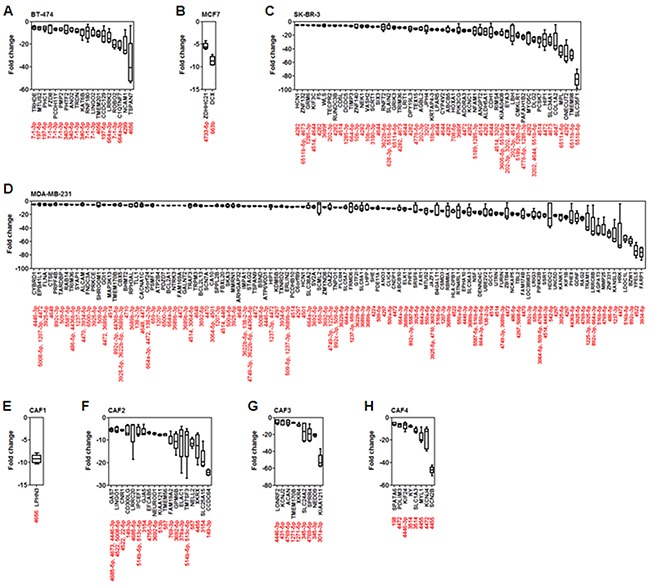
Analysis of fold change in expression level of putative target mRNAs of compression-upregulated microRNAs Compression-induced decrease in expression levels of microRNA target genes in **A.** BT-474, **B.** MCF7, **C.** SK-BR-3, **D.** MDA-MB-231, **E.** CAF1, **F.** CAF2, **G.** CAF3, and **H.** CAF4 cells. Compression-induced target-gene downregulation was analyzed in the same samples of total RNA used for microRNA array analysis. Target mRNA downregulation showing more than 5-fold change on average was presented for analysis.

The compression-upregulated microRNAs are associated with a large number of target genes, as shown in Supplementary Figure S1. The primary function of microRNA is to suppress target mRNA expression. Therefore, the putative target genes of the compression-upregulated microRNAs, which have more than 80 target prediction score (Supplementary Table S2) from miRDB (http://mirdb.org/miRDB), and the fold change values of the putative target mRNAs were analyzed by mRNA array using the same samples of total RNA used for microRNA array. Greater than 5-fold decrease was used as the cut-off for target mRNA downregulation by microRNAs. In BT-474 cells, 19 target mRNAs were downregulated. Of these, eight were potential targets of miR-7-1-3p (TRHDE, FZD8, PCDH11Y, PMP2, TRDN, RNF180, LINGO2, and ROBO2). The most downregulated target mRNA was TSPAN7, a putative target of miR-4656 (Figure 3A). In MCF7 cells, ZDHHC21 (a putative target of miR-4733-5p) and DCX (a putative target of miR-663b) were downregulated (Figure 3B). SK-BR-3 cells showed downregulation of 53 target mRNAs. SLC35F1 (a putative target of miR-551b-5p) was downregulated by approximately 80-fold. MPL (a putative target of miR6511a-5p), ONECUT2 (a putative target of miR-4282), and TMEM98 (a putative target of miR-6511b-5p) were downregulated by approximately 40-fold (Figure 3C). In MDA-MB-231 cells, 114 target mRNAs were downregulated. YPEL5 (a putative target of miR-4522) and FARP1 (a putative target of miR-3934-5p) were downregulated by approximately 80-fold. Target mRNAs downregulated by more than 40-fold were TRAM1 (a putative target of miR-1225-3p and 3925-5p), LRRC8B (a putative target of miR-892c-3p and miR-664a-3p), AGPAT3 (a putative target of miR-516b-5p), MMP25 (a putative target of miR-4769-5p), ZNF331 (a putative target of miR-486-5p), KANSL3 (a putative target of miR-1237-3p), HRK (a putative target of miR-1237-3p), and LDOC1L (a putative target of miR-516b-5p) (Figure 3D). In CAF1 cells, LPHN3 (a putative target of miR-4656) was downregulated by approximately 10-fold (Figure 3E). The largest number of downregulated target mRNAs (19) was seen in CAF2 cells; CCDC64 (a putative target of miR-149-3p) was downregulated to the greatest extent (by approximately 25-fold) in these cells. FAM19A2 (a putative target of miR-769-3p), GPM6B (a putative target of miR-3692-5p), ELAC1 (a putative target of miR-557), XKRX (a putative target of miR-4455), and SLC25A15 (a putative target of miR-3154) were downregulated by 10- to 20-fold (Figure 3F). In CAF3 cells, nine target mRNAs were downregulated. KIAA1211 (a putative target of miR-301a-3p) was downregulated by more than 50-fold. SLC24A2 (a putative target of miR-345-3p), SPRR4 (a putative target of miR-4769-5p), and NEDD9 (a putative target of miR-345-3p) were downregulated by 10-fold (Figure 3G). CAF4 cells showed downregulation of eight target mRNAs. SCN2B (a putative target of miR-4455) was downregulated by more than 40-fold. MYL1 (a putative target of miR-4455) and KCNJ4 (a putative target of miR-4472) were downregulated by approximately 20-fold (Figure 3H).

### Analysis of relationship of compression-upregulated microRNA target genes to tumor progression

**Figure 4:**
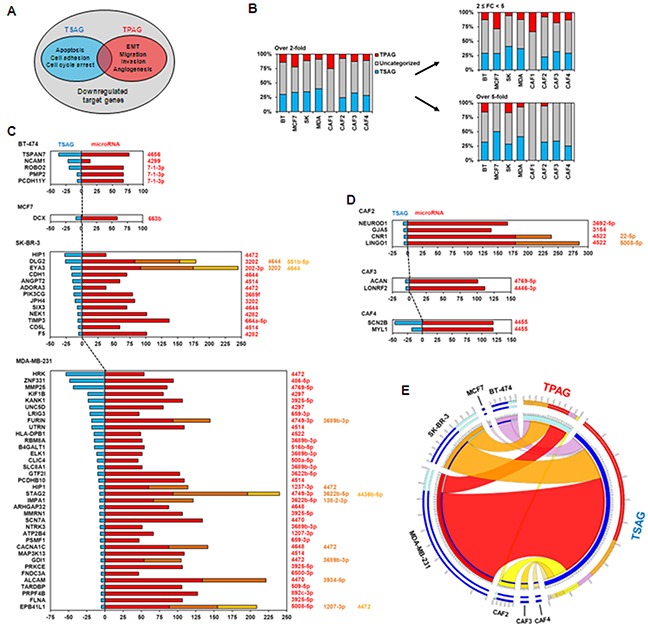
Analysis of relationship between compression-upregulated microRNAs and downregulated target genes **A.** Venn diagram of tumor suppression-associated genes (TSAGs) and tumor promotion-associated genes (TPAGs) among compression-downregulated target genes. TSAGs include the genes associated with apoptosis, cell adhesion, and cell cycle arrest. TPAGs include the genes related to epithelial-mesenchymal transition (EMT), migration, invasion, and angiogenesis. The intersection between TSAGs and TPAGs was excluded to avoid confusion. **B.** Proportional analysis of TSAGs and TPAGs in downregulated putative target genes of compression-upregulated microRNAs. The intersection between TSAGs and TPAGs was excluded to avoid confusion. BT, BT-474 cells; SK, SK-BR-3 cells; MDA, MDA-MB-231 cells. Inverse correlation between expression of microRNAs and their target genes in **C.** breast cancer cell lines and **D.** CAFs. Average expression values of microRNAs and target genes were analyzed. Colors of microRNAs are matched to those of bars in graph. **E.** Correlation analysis between the downregulated target genes of compression-upregulated microRNAs, TSAGs, and TPAGs. Circos software (version 0.63-9) [49] was used for analysis. Target mRNAs showing upregulation by more than 5-fold on average were used in analysis.

Compression induced the upregulation of microRNAs in breast cancer cell lines and CAFs, coupled with downregulation of putative target mRNAs of the microRNAs (Figures 1–3). However, it still remains unknown whether compression-induced microRNA upregulation is associated with tumor progression. To investigate the relationship between compression-mediated upregulation of microRNAs and tumor progression, the proportions of tumor suppression-associated genes (TSAGs, the genes involved in apoptosis, cell adhesion, and cell cycle arrest) and tumor promotion-associated genes (TPAGs, the genes involved in epithelial-mesenchymal transition [EMT], migration, invasion, and angiogenesis) were analyzed among the target genes of compression-upregulated microRNAs (Figure 4A). Because the primary function of microRNAs is to suppress expression of mRNAs, upregulation of microRNAs targeting TPAGs may suppress tumor progression, whereas upregulation of microRNAs targeting TSAGs may contribute to tumor progression by blocking apoptosis, cell adhesion, and cell cycle arrest. As shown in Figure 4B, among target genes of compression-upregulated microRNAs, TSAGs represented more than 25% of genes in all cell types except CAF1, whereas TPAGs generally represented less than 15% at the fold change cut-off value of 2. At the cut-off value of 5, the proportion of TSAGs averaged 30%, whereas TPAGs averaged less than 5% in most cells, with the exception of BT-474 and SK-BR-3 cells. Among the breast cancer cell lines, MDA-MB-231 showed the largest number of TSAGs, and MCF-7 showed the smallest. TSAGs with greater than 25-fold downregulation were TSPAN7 (a putative target of miR4656) in BT-474 cells; HIP1 (a putative target of miR-4472) and DLG2 (a putative target of miR-3202, miR-4644, and miR-551b-5p) in SK-BR-3 cells; and HRK (a putative target of miR-4472), ZNF331 (a putative target of miR-486-5p), and MMP25 (a putative target of miR-4769-5p) in MDA-MB-231 cells (Figure 4C). CAFs did not show many downregulated TSAGs compared to the breast cancer cell lines. There were none in CAF1 cells, four in CAF2 cells, and two each in CAF3 and CAF4 cells. The TSAG with greater than 25-fold downregulation was SCN2B (a putative target of miR-4455) in CAF4 cells (Figure 4D). TSAGs and TPAGs for each cell type are listed in Supplementary Table S3.

## DISCUSSION

MicroRNA array analysis showed that alteration of microRNA expression level was induced by compression, and to a greater extent in breast cancer cell lines than CAFs. Interestingly, the major alteration of compression-induced microRNA expression was upregulation, not downregulation (Figure 1B–1E). To investigate whether compression-induced microRNA upregulation contributes to tumor progression, fold change in levels of putative target mRNAs of compression-upregulated microRNAs was analyzed in a parallel mRNA array using the same samples of total RNA used for microRNA array. Next, downregulated putative target mRNAs were classified as TSAGs and TPAGs because the primary function of microRNA is to suppress target gene expression [30, 31]. As shown in Figure 4B, the portion of TSAGs was much higher than that of TPAGs. It was previously reported that microRNA-mediated downregulation of TSAGs leads to tumor progression; miR-21 overexpression contributed to hepatocellular cancer growth and spreading via downregulation of PTEN [10]. Pdcd4 suppression by miR-21 induced invasion, intravasation, and metastasis of colorectal cancer cell lines in chicken-embryo-metastasis assay [32]. MicroRNA let-7 suppressed proliferation in a lung cancer cell line via HMGA2 suppression [33].

MDA-MB-231 cells showed the largest number of compression-upregulated microRNAs (Figure 2D) and downregulated TSAGs (Figure 4E and Supplementary Figure S2). Manipulation of levels of microRNA expression specific to MDA-MB-231 cells may be a therapeutic option for incurable cancers. In a mouse model of lymphoma, miR-155 antisense RNA slowed the growth of pre–B-cell tumors [34]. Ectopic expression of miR-133b inhibited the growth of colorectal cancer cells (SW-620) in an in vivo tumor xenograft model [35]. Restoration of miR-34 expression in pancreatic cancer cell line MiaPaCa2 by transfection inhibited clonogenic cell growth and invasion, induced apoptosis and cell cycle arrest, and sensitized cells to chemo- and radiotherapy in vivo [36]. MCF-7 and BT-474 showed less number of compression-upregulated microRNA than SK-BR-3 and MDA-MB-231 (Figure 2A–2D). These heterogeneous responses of breast cancer cells to compression may be caused by different expression status of growth factor receptors such as ER, PR, and Her2 since membrane-anchored receptors can function as mechanosensors [37]. It was previously reported that ER plays an important role in the mechanotransduction of osteocyte and osteoblasts [38]. As another example, angiotensin II type 1 receptor is known to be activated by mechanical stress without the involvement of angiotensin II [39, 40].

Targeting microRNAs expressed in cells of the tumor microenvironment may be a good approach. Overexpression of miR-148a in CAFs inhibited the migration of cells of five endometrial cancer lines [41]. Reconstitution of miR-15 and miR-16 impaired the tumor-supportive capability of CAFs [42]. However, compression-induced alteration of microRNA expression was different between CAFs, which may be caused by inter-individual heterogeneity of normal stroma [43]. In spite of inter-individual heterogeneity of CAF, targeting a compression-upregulate microRNA on CAF may be an effective cancer therapy. As shown in Figure 4B, over 25% of compression-upregulated microRNAs in CAF2, 3, and 4 are predicted to target tumor suppressor genes (TSGs). TSG inactivation of CAF is often observed in human cancer and increases paracrine signaling to tumor cells via cytokine production [44]. miR-4769-5p and miR-4446-3p were upregulated by compression in MDA-MB-231 cells and CAFs (Figure 2I). Communication between a tumor and its microenvironment is crucial for tumor progression [45]. Therefore, miR-4769-5p and miR-4446-3p may be effective therapeutic targets for incurable cancers because they simultaneously downregulate TSAG expression in both cancer cells and CAFs, a major component of the tumor microenvironment.

In this study, we present the microRNA transcriptome-wide analysis of compression-induced changes in microRNA expression levels in breast cancer cell lines and CAFs treated with different degrees of compression. In microRNA array analysis, alteration of microRNA expression level was induced by compression, and to a greater extent in breast cancer cell lines than CAFs. The major alteration of compression-induced microRNA expression was upregulation. MDA-MB-231 cells were the most susceptible to compression in terms of changes in microRNA expression level. Finally, miR-4769-5p and miR4446-3p may be effective therapeutic targets for triple negative breast cancer.

## MATERIALS AND METHODS

### Tissue acquisition

Human breast tumor tissues were obtained from four invasive ductal carcinoma (IDC) patients who had surgery at Severance Hospital of the Yonsei University Health System, South Korea. All patients donating the tissues were informed of tissue use for comprehensive experiments on breast cancer and provided written informed consent. The research protocol was approved by the Severance Hospital Ethics Committee (IRB number 4-2008-0383).

### Isolation of CAFs and cell cultures

CAFs were isolated as previously described [46]. Briefly, IDC tissues were minced and then digested overnight in a collagenase cocktail (ISU ABXIS; Seoul, South Korea). Digested tissues were filtered through a 70-μm cell strainer (SPL Life Science; Pocheon-si, South Korea). Cells were separated using Ficoll gradients, washed with phosphate-buffered saline (PBS), resuspended in Dulbecco's Modified Eagle medium (DMEM)/F12 medium containing 20% (v/v) fetal bovine serum (FBS), 100 IU/mL penicillin, and 100 μg/mL streptomycin (Gibco BRL; Grand Island, NY, USA), and cultured at 37°C in a humidified atmosphere of 5% CO_2_. The fibrotic characteristics of the isolated cells were confirmed by microscopic examination of morphology and immunofluorescence analysis using antibodies against vimentin (Abcam; Cambridge, UK), cytokeratin (Dako; Glostrup, Denmark), and cytokeratin 5 (Novocastra; Newcastle upon Tyne, UK).

### Compression assay and sample preparation

For three-dimensional culture and convenient sample preparation, cells were cultured in alginate beads. To prepare alginate beads containing cells, pellets of BT-474, MCF7, SK-BR-3, and MDA-MB-231 cells and CAFs were resuspended in 0.5% alginate solution (Sigma-Aldrich; Missouri, USA) at a density of 5 × 10^6^ cells/mL. Cells suspended in alginate were added dropwise into gently stirred 102 mM CaCl_2_solution for polymerization, using a syringe with a 21-gauge needle [47]. The cell-alginate beads were washed with PBS 2–3 times and cultured at 37°C in a humidified atmosphere of 5% CO_2_ for 24 h. During this period, the cells stabilized and extracellular matrix was deposited around the cells. For compression assay, pre-cultured cell-alginate beads were embedded in 2% low-melting agarose, equilibrated in medium for 1 h, and then relative compression units (RCUs) of 0, 0.5, 1, 2, 5, or 10 were applied for 24 h using the cube filled with iron beads (Supplementary Figure S3). One RCU equals 5.8 mmHg (∼0.773 kPa), which is the approximate compression value of a native tumor microenvironment [23]. An empty cube was loaded on agarose-scaffolded alginate beads for the controls (0RCU). Compression force transfer to cells via alginate beads and agarose gel was evaluated as deformation at 24 h. Uniaxial deformation to 50% was observed in alginate beads and agarose gel. The diffusion rate of nutrients can alter gene expression [48]. Since molecular weight of almost all nutrients in the media used in our study was below 500 Da, nutrient diffusion rate was indirectly investigated in 2% agarose gel at different RCUs using Ponceau S (approximately 760 Da). The diffusion rate was not different in 2% agarose gel compressed at different RCUs and in uncompressed 4% agarose gel, which has similar pore sizes to those of 50%-deformed 2% agarose gel. To regain cells from the alginate beads, beads were depolymerized with 55 mM EDTA [47], washed with PBS twice, and centrifuged at 250g for 3 min. The cell pellets were used immediately or stored at -80°C.

### MicroRNA and mRNA arrays

For simultaneous isolation of microRNA and mRNA from the same samples, total RNA was extracted from uncompressed and compressed BT-474, MCF7, SK-BR-3, MDA-MB-231 cells and CAFs using Trizol^®^ (Invitrogen Life Technologies; Grand Island, NY, USA). RNA quality was assessed using an Agilent 2100 Bioanalyzer (Agilent Technologies; Santa Clara, CA, USA), and total RNA (OD_260_/OD_230_ >1.8; 28s/18s of 1.5; and RNA integrity number [RIN] of 7.0) was analyzed on the human microRNA array (Release 19.0, Agilent Technologies) and the SurePrint G3 Human Gene expression 8×60K v2 Microarray (Agilent Technologies). Fold change values were calculated as the expression level of a gene at each RCU to that at an RCU of 0 (control without compression).

## SUPPLEMENTARY FIGURES AND TABLES






