# Supplementary material for: Transcriptome-wide analysis of compression-induced microRNA expression alteration in breast cancer for mining therapeutic targets
Source: Oncotarget. 2016 Mar 24;7(19):27468–78. doi: 10.18632/oncotarget.8322 (PMC5053664; doi:10.18632/oncotarget.8322)
Supplement: Supplementary file 1 [file oncotarget-07-27468-s001.pdf]

SUPPLEMENTARY FIGURES AND TABLES

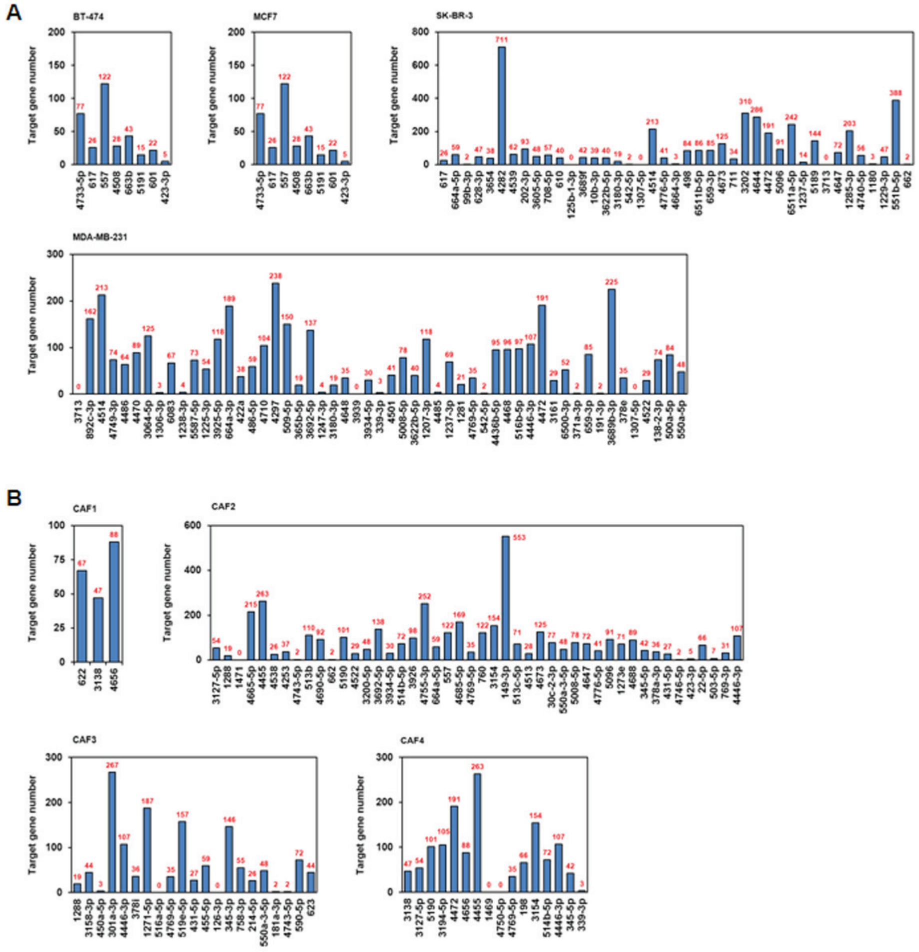

Supplementary Figure S1: Numbers of putative target genes of compression-upregulated microRNAs. Numbers of putative target genes in **A.** breast cancer cell lines and **B.** CAFs. Target genes having prediction score >80 were selected from miRDB (http://mirdb.org/miRDB/).

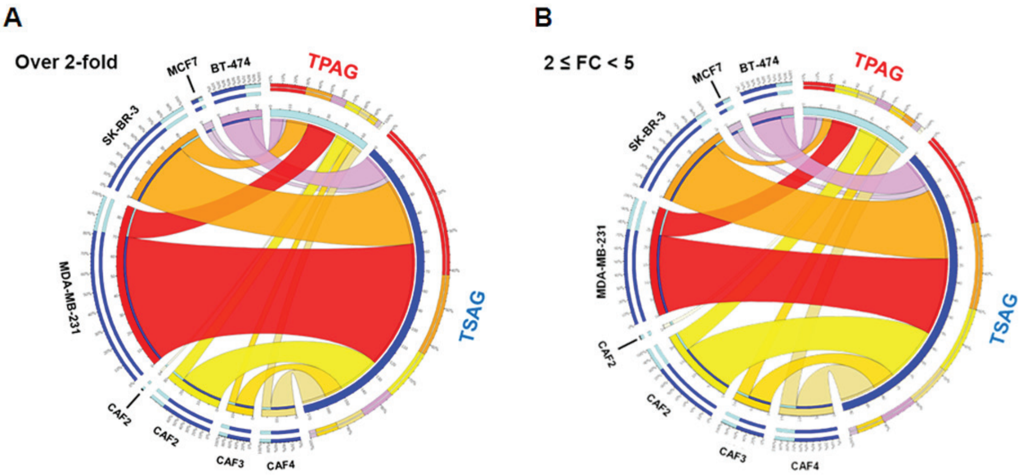

Supplementary Figure S2: Correlation analysis between downregulated target genes of compression-upregulated microRNAs, TSAGs, and TPAGs at different cut-off values. Correlation analysis between downregulated putative target genes, TSAGs, and TPAGs at the cut-off values **A.** of 2 (>2-fold) and **B.** between 2 and 5 (2 ≤ fold change (FC) < 5) in breast cancer cell lines and CAFs using Circos software (version 0.63-9).

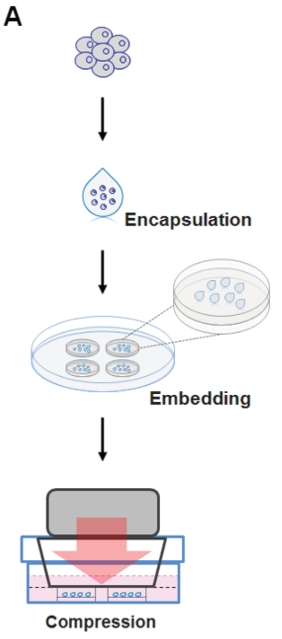

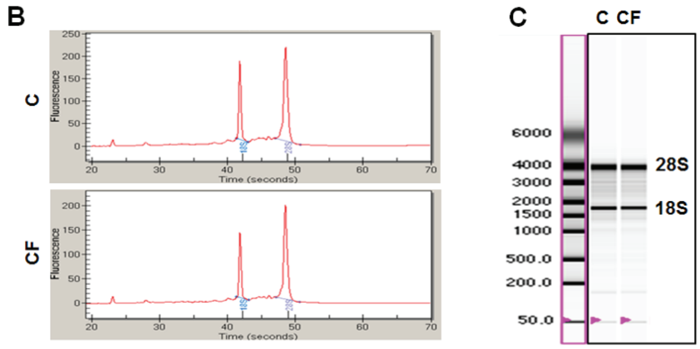

D

Sample	$OD_{260/280}$	$OD_{260/230}$	Total (μg)	Ratio(28s/18s)	RIN
BT-474	1.87	1.63	5.2579	2.0	7.7
MCF7	1.89	0.52	4.8480	1.9	8.0
SK-BR-3	1.87	1.34	6.2153	1.7	7.4
MDA-MB-231	1.85	1.66	4.9849	1.9	7.7
CAF	1.88	0.53	4.6263	1.9	8.3

Supplementary Figure S3: Agarose-scaffolded alginate bead culture for 3D static compression model and RNA quality validation. A. Scheme of agarose-scaffolded alginate bead culture for 3D static compression model. Cells were encapsulated with alginate (cell-alginate bead formation) and pre-cultured for 1 day. The alginate beads containing cells were embedded into agarose scaffold, equilibrate for 1 hour, and then statically compressed at different compression conditions for 1 day. For compression, the cube filled with iron beads was loaded onto agarose-scaffolded alginate beads. Empty cube was used for control. **B.** RNA electropherogram (peak pattern) and **C.** RNA electrophoretic trace (migration pattern) of control (uncompressed) and compressed cells. **D.** Quality control result of compressed cells. After compression at 1RCU (5.8 mmHg) for 1 day, the cells were recovered by depolymerizing alginate beads with 40 mM EDTA and then lysed to extract total RNA. The quality of RNA was evaluated by using an Agilent 2100 Bioanalyzer system.

Supplementary Table S1: MicroRNAs commonly upregulated in breast cancer cell lines and CAFs at all RCUs.

See Supplementary File 1

Supplementary Table S2: Putative target genes of compression-upregulated microRNAs.

See Supplementary File 2

Supplementary Table S3: Tumor suppression-associated genes (TSAGs) and tumor promotion-associated genes (TPAGs)

Cell	Tumor Suppression-Associated Genes (TSAGs)	Tumor Promotion-Associated Genes (TPAGs)
BT-474	DLG2 HIVEP3 DYNC1I1 CALCR PTPRC NCAM2 EMILIN2 MAT1A AKR1B1 PCDH11Y PMP2 LRRK2 ROBO2 NCAM1 TSPAN7	SLC12A5 GRB14 FGL2 ARL13B TRHDE FZD8 TMEM201
MCF7	TARDBP PLEKHA8 DCX	TET1 TBX1
SK-BR-3	PROS1 PPP2R2B LAMA1 PPARGC1B SERPINB3 MSTN PLAA MAP1A AGTR1 ERCC4 SIM2 CAPZB TWIST1 GIMAP4 SDC3 OGN PFKFB2 SIRPA GABRB3 LEP CUL3 TAT F5 CD5L TIMP3 NEK1 SIX3 JPH4 HOXA1 PIK3CG ADORA3 ANGPT2 CDH1 EYA3 DLG2 HIP1 COL1A2	SHC2 HOXB9 BMPER RUNDC3B VASH2 SCRT1 LPAR5 ABCB5 KCNC1 MYO5C MPL ONECUT2
MDA-MB-231	TIE1 PRNP COL19A1 EIF4G3 KPNA4 NTSR1 HSPA8 FLT1 PKD1 CYB5R3 EFNA3 IL1RL1 RPS6KB1 FAF2 DNM3 ERCC4 CD247 DCLK1 SORBS2 PADI2 LPP MSI2 PLAGL2 ADORA3 EPB41L1 FLNA PRPF4B TARDBP RAB14 ALCAM FNDC3A PRKCE GDI1 MAP3K13 CACNA1C PSMF1 ATP2B4 BCL2A1 NTRK3 TRAF3 BCL2L13 SCN7A MMRN1 ARHGAP32 IMPA1 STAG2 HIP1 TXNRD2 ERLIN2 PCDHB10 TNPO1 PLCB4 FRMD6 GTF2I SLC8A1 CLIC4 ELK1 JAZF1 B4GALT1 RBM8A HLA-DPB1 UTRN FURIN LRIG3 UNC5D KANK1 KIF1B GDNF MMP25 ZNF331 HRK	SERBP1 PPFIA1 BCAR3 APOBEC3B ANKRD28 RAB11A NAV2 APOBEC3D CTSE CBX5 GALNT2 KDM5B SLC38A2 SV2A ZMYND8 RTN4RL1
CAF1		PHF20L1
CAF2	PCDH9 KCNJ5 PPT1 FABP4 NAMPT PBX1 ALDH1A2 EMILIN2 HOXC4 LOXL1 FHL1 PCDHGA8 GATA4 SEL1L HUS1 APITD1 MAPK9 GAS7 LINGO1 CNR1 GJA5 NEUROD1 NELL2	MYO1D STARD13 SLC48A1 TSPAN2 TMEM201 IFNAR1 IPCEF1
CAF3	MEF2C ABCC5 UNC5D SLC39A1 GAS7 FRAT1 DLL1 LONRF2 ACAN NEDD9	SLC12A7 MFAP3L NEDD4L ETV1
CAF4	COL19A1 LONRF2 PBX1 SYT1 CXCL9 ANKRD1 NTSR1 PDCD1LG2 BCL11B ATP8A1 SEMA4G MYL1 SCN2B	IPCEF1 SLC48A1 RNGTT TMEM201 ABCG4
