# Supplementary material for: Transcriptome-wide analysis of compression-induced microRNA expression alteration in breast cancer for mining therapeutic targets
Source: Oncotarget. 2016 Mar 24;7(19):27468–78. doi: 10.18632/oncotarget.8322 (PMC5053664; doi:10.18632/oncotarget.8322)
Supplement: Supplementary file 2 [file oncotarget-07-27468-s002.docx]

**Supplementary Table S1. MicroRNAs commonly upregulated in breast cancer cell lines and CAFs at all RCUs.**

A) BT-474

| Fold change | microRNA | Relative compression unit | | | | | Accession No. |
| --- | --- | --- | --- | --- | --- | --- | --- |
|  |  | 0.5 | 1 | 2 | 5 | 10 |  |
| > 10 | hsa-miR-671-5p | 164.783 | 180.571 | 165.303 | 68.267 | 184.968 | MIMAT0003880 |
|  | hsa-miR-4486 | 161.783 | 173.679 | 164.599 | 121.691 | 188.167 | MIMAT0019020 |
|  | hsa-miR-664b-5p | 140.419 | 160.629 | 165.414 | 71.670 | 171.599 | MIMAT0022271 |
|  | hsa-miR-484 | 89.218 | 60.566 | 118.144 | 61.001 | 109.603 | MIMAT0002174 |
|  | hsa-miR-664a-3p | 87.581 | 84.496 | 116.086 | 54.826 | 84.938 | MIMAT0005949 |
|  | hsa-miR-4656 | 82.017 | 83.968 | 66.935 | 62.751 | 89.175 | MIMAT0019723 |
|  | hsa-miR-340-5p | 80.150 | 80.471 | 84.331 | 57.662 | 76.427 | MIMAT0004692 |
|  | hsa-miR-1249 | 73.194 | 37.472 | 137.405 | 132.255 | 78.051 | MIMAT0005901 |
|  | hsa-miR-7-1-3p | 51.931 | 84.639 | 60.701 | 58.644 | 83.563 | MIMAT0004553 |
|  | hsa-miR-1281 | 41.547 | 40.301 | 94.381 | 56.302 | 44.221 | MIMAT0005939 |
|  | hsa-miR-4299 | 15.770 | 15.359 | 13.092 | 10.892 | 15.060 | MIMAT0016851 |
|  | hsa-miR-197-5p | 11.029 | 10.587 | 11.496 | 8.910 | 10.806 | MIMAT0022691 |
| 10 ≥ >2 | hsa-miR-4672 | 5.880 | 6.508 | 7.031 | 5.704 | 6.437 | MIMAT0019754 |
|  | hsa-miR-4690-5p | 4.616 | 4.728 | 3.966 | 2.447 | 5.164 | MIMAT0019779 |
|  | hsa-miR-1268a | 4.082 | 3.680 | 4.275 | 3.576 | 4.052 | MIMAT0005922 |
|  | hsa-miR-4507 | 4.038 | 4.170 | 4.491 | 3.422 | 4.385 | MIMAT0019044 |
|  | hsa-miR-1587 | 3.377 | 3.521 | 3.571 | 2.710 | 3.639 | MIMAT0019077 |
|  | hsa-miR-424-3p | 3.317 | 3.118 | 2.934 | 3.014 | 3.559 | MIMAT0004749 |
|  | hsa-miR-4505 | 3.049 | 3.336 | 3.365 | 2.573 | 3.356 | MIMAT0019041 |
|  | hsa-miR-4532 | 2.958 | 3.196 | 3.109 | 2.462 | 3.356 | MIMAT0019071 |
|  | hsa-miR-4530 | 2.874 | 3.197 | 3.208 | 2.272 | 3.139 | MIMAT0019069 |
|  | hsa-miR-4430 | 2.342 | 2.811 | 2.707 | 2.302 | 2.964 | MIMAT0018945 |
|  | hsa-miR-3656 | 2.221 | 2.553 | 2.597 | 2.000 | 2.643 | MIMAT0018076 |
|  | hsa-miR-1275 | 2.150 | 2.901 | 2.813 | 2.072 | 3.128 | MIMAT0005929 |
|  | hsa-miR-4459 | 2.104 | 3.234 | 3.228 | 2.650 | 3.813 | MIMAT0018981 |

B) MCF7

| Fold change | microRNA | Relative compression unit | | | | | Accession No. |
| --- | --- | --- | --- | --- | --- | --- | --- |
|  |  | 0.5 | 1 | 2 | 5 | 10 |  |
| > 10 | hsa-miR-4733-5p | 132.786 | 137.545 | 151.908 | 153.724 | 58.186 | MIMAT0019857 |
|  | hsa-miR-617 | 98.146 | 120.944 | 142.791 | 143.307 | 109.831 | MIMAT0003286 |
|  | hsa-miR-557 | 91.123 | 109.728 | 113.852 | 146.967 | 91.182 | MIMAT0003221 |
|  | hsa-miR-3622a-5p | 78.983 | 93.995 | 117.263 | 106.908 | 73.094 | MIMAT0018003 |
|  | hsa-miR-4508 | 56.158 | 65.639 | 97.114 | 96.786 | 54.178 | MIMAT0019045 |
|  | hsa-miR-663b | 52.182 | 56.777 | 57.177 | 64.952 | 62.396 | MIMAT0005867 |
|  | hsa-miR-5191 | 40.432 | 116.134 | 122.739 | 111.363 | 92.957 | MIMAT0021122 |
|  | hsa-miR-601 | 21.613 | 77.396 | 117.342 | 108.826 | 48.484 | MIMAT0003269 |
|  | hsa-miR-423-3p | 20.467 | 44.990 | 54.288 | 39.640 | 42.727 | MIMAT0001340 |
| 10 ≥ >2 | hsa-miR-550b-2-5p | 4.383 | 4.973 | 5.982 | 6.260 | 4.034 | MIMAT0022737 |
|  | hsa-miR-197-5p | 4.205 | 4.869 | 5.427 | 5.294 | 5.197 | MIMAT0022691 |
|  | hsa-miR-4299 | 3.845 | 4.601 | 7.272 | 6.319 | 4.904 | MIMAT0016851 |
|  | hsa-miR-3156-5p | 3.631 | 4.828 | 5.290 | 5.299 | 3.047 | MIMAT0015030 |
|  | hsa-miR-1288 | 3.609 | 4.886 | 5.467 | 5.522 | 3.357 | MIMAT0005942 |
|  | hsa-miR-4788 | 3.427 | 3.987 | 4.226 | 4.156 | 3.081 | MIMAT0019958 |
|  | hsa-miR-3917 | 3.337 | 5.076 | 5.597 | 5.246 | 4.249 | MIMAT0018191 |
|  | hsa-miR-6132 | 3.094 | 3.875 | 4.659 | 4.284 | 4.282 | MIMAT0024616 |
|  | hsa-miR-6127 | 3.027 | 3.582 | 4.125 | 4.036 | 3.597 | MIMAT0024610 |
|  | hsa-miR-149-3p | 3.008 | 3.408 | 3.774 | 3.954 | 2.978 | MIMAT0004609 |
|  | hsa-miR-4430 | 2.991 | 3.537 | 3.536 | 3.626 | 3.419 | MIMAT0018945 |
|  | hsa-miR-4497 | 2.652 | 2.790 | 2.841 | 2.924 | 3.411 | MIMAT0019032 |
|  | hsa-miR-4478 | 2.603 | 3.201 | 3.487 | 3.510 | 2.480 | MIMAT0019006 |
|  | hsa-miR-874 | 2.585 | 3.148 | 3.368 | 3.242 | 2.982 | MIMAT0004911 |
|  | hsa-miR-1224-5p | 2.513 | 2.750 | 2.775 | 2.876 | 3.028 | MIMAT0005458 |
|  | hsa-miR-4486 | 2.455 | 2.921 | 3.010 | 2.968 | 2.771 | MIMAT0019020 |
|  | hsa-miR-1914-3p | 2.454 | 3.011 | 3.560 | 3.520 | 2.302 | MIMAT0007890 |
|  | hsa-miR-6087 | 2.398 | 2.495 | 2.659 | 2.583 | 3.061 | MIMAT0023712 |
|  | hsa-miR-4257 | 2.338 | 2.954 | 3.425 | 3.181 | 2.983 | MIMAT0016878 |
|  | hsa-miR-1249 | 2.337 | 2.710 | 2.285 | 2.448 | 4.214 | MIMAT0005901 |
|  | hsa-miR-6131 | 2.290 | 2.781 | 3.836 | 3.703 | 2.314 | MIMAT0024615 |
|  | hsa-miR-3620-5p | 2.272 | 2.706 | 2.555 | 2.612 | 2.208 | MIMAT0022967 |
|  | hsa-miR-762 | 2.270 | 2.520 | 2.668 | 2.726 | 2.878 | MIMAT0010313 |
|  | hsa-miR-6075 | 2.268 | 3.158 | 3.332 | 3.270 | 3.051 | MIMAT0023700 |
|  | hsa-miR-1587 | 2.250 | 2.645 | 3.031 | 2.923 | 2.852 | MIMAT0019077 |
|  | hsa-miR-1268a | 2.238 | 2.405 | 2.648 | 2.641 | 2.858 | MIMAT0005922 |
|  | hsa-miR-1225-5p | 2.213 | 2.457 | 2.667 | 2.595 | 2.609 | MIMAT0005572 |
|  | hsa-miR-4507 | 2.205 | 2.519 | 2.679 | 2.591 | 2.692 | MIMAT0019044 |
|  | hsa-miR-4721 | 2.171 | 2.496 | 2.831 | 2.829 | 2.742 | MIMAT0019835 |
|  | hsa-miR-4728-5p | 2.157 | 2.671 | 3.185 | 3.191 | 2.263 | MIMAT0019849 |
|  | hsa-miR-494 | 2.140 | 2.345 | 2.516 | 2.829 | 2.495 | MIMAT0002816 |
|  | hsa-miR-4530 | 2.139 | 2.126 | 2.227 | 2.217 | 2.402 | MIMAT0019069 |
|  | hsa-miR-2392 | 2.120 | 2.168 | 2.458 | 2.379 | 2.376 | MIMAT0019043 |
|  | hsa-miR-3198 | 2.118 | 2.678 | 3.441 | 3.364 | 2.086 | MIMAT0015083 |
|  | hsa-miR-642b-3p | 2.077 | 2.162 | 2.204 | 2.317 | 2.514 | MIMAT0018444 |
|  | hsa-miR-1275 | 2.077 | 2.331 | 2.498 | 2.493 | 2.871 | MIMAT0005929 |
|  | hsa-miR-4672 | 2.072 | 2.590 | 2.943 | 2.914 | 2.331 | MIMAT0019754 |
|  | hsa-miR-4669 | 2.061 | 2.414 | 2.715 | 2.755 | 2.235 | MIMAT0019749 |
|  | hsa-miR-1233-1-5p | 2.049 | 2.420 | 2.577 | 2.496 | 2.096 | MIMAT0022943 |
|  | hsa-miR-6076 | 2.035 | 2.182 | 2.517 | 2.549 | 2.151 | MIMAT0023701 |
|  | hsa-miR-4462 | 2.033 | 2.497 | 2.575 | 2.839 | 2.370 | MIMAT0018986 |
|  | hsa-miR-4505 | 2.028 | 2.365 | 2.836 | 2.725 | 2.695 | MIMAT0019041 |
|  | hsa-miR-6510-5p | 2.025 | 2.240 | 2.615 | 2.661 | 2.673 | MIMAT0025476 |
|  | hsa-miR-760 | 2.018 | 2.336 | 2.214 | 2.734 | 2.355 | MIMAT0004957 |
|  | hsa-miR-5006-5p | 2.008 | 2.260 | 2.318 | 2.378 | 2.283 | MIMAT0021033 |

C) SK-BR-3

| Fold change | microRNA | Relative compression unit | | | | | Accession No. |
| --- | --- | --- | --- | --- | --- | --- | --- |
|  |  | 0.5 | 1 | 2 | 5 | 10 |  |
| > 10 | hsa-miR-617 | 126.100 | 171.262 | 191.339 | 165.702 | 180.517 | MIMAT0003286 |
|  | hsa-miR-664a-5p | 117.113 | 144.940 | 152.750 | 123.584 | 145.548 | MIMAT0005948 |
|  | hsa-miR-99b-3p | 113.122 | 166.323 | 155.136 | 160.710 | 152.311 | MIMAT0004678 |
|  | hsa-miR-628-3p | 98.754 | 136.800 | 162.767 | 121.745 | 137.553 | MIMAT0003297 |
|  | hsa-miR-3654 | 98.393 | 128.811 | 173.023 | 112.888 | 159.989 | MIMAT0018074 |
|  | hsa-miR-4282 | 93.582 | 85.909 | 123.757 | 85.252 | 118.047 | MIMAT0016912 |
|  | hsa-miR-4539 | 88.745 | 36.559 | 92.830 | 65.423 | 80.317 | MIMAT0019082 |
|  | hsa-miR-202-3p | 82.964 | 93.479 | 97.800 | 74.740 | 109.949 | MIMAT0002811 |
|  | hsa-miR-3605-5p | 82.500 | 132.128 | 155.026 | 127.063 | 138.192 | MIMAT0017981 |
|  | hsa-miR-708-5p | 81.844 | 105.775 | 120.443 | 112.603 | 120.297 | MIMAT0004926 |
|  | hsa-miR-610 | 80.477 | 57.550 | 88.399 | 50.632 | 93.083 | MIMAT0003278 |
|  | hsa-miR-125b-1-3p | 79.021 | 113.115 | 122.017 | 110.611 | 121.280 | MIMAT0004592 |
|  | hsa-miR-3689f | 76.136 | 37.239 | 106.865 | 73.073 | 102.000 | MIMAT0019010 |
|  | hsa-miR-10b-3p | 75.960 | 83.494 | 107.457 | 101.958 | 100.601 | MIMAT0004556 |
|  | hsa-miR-3622b-5p | 73.641 | 95.284 | 109.695 | 80.598 | 100.110 | MIMAT0018005 |
|  | hsa-miR-3180-3p | 71.284 | 118.890 | 124.970 | 124.952 | 118.209 | MIMAT0015058 |
|  | hsa-miR-542-5p | 70.878 | 105.845 | 109.143 | 96.346 | 109.152 | MIMAT0003340 |
|  | hsa-miR-1307-5p | 68.981 | 38.880 | 89.755 | 77.848 | 89.989 | MIMAT0022727 |
|  | hsa-miR-4514 | 68.526 | 38.622 | 79.544 | 32.761 | 76.459 | MIMAT0019051 |
|  | hsa-miR-4776-5p | 65.906 | 36.252 | 84.297 | 61.020 | 82.041 | MIMAT0019932 |
|  | hsa-miR-4664-3p | 65.272 | 90.044 | 98.537 | 86.115 | 92.914 | MIMAT0019738 |
|  | hsa-miR-498 | 60.509 | 36.185 | 78.333 | 65.101 | 72.272 | MIMAT0002824 |
|  | hsa-miR-6511b-5p | 51.093 | 66.806 | 80.546 | 62.226 | 67.031 | MIMAT0025847 |
|  | hsa-miR-659-3p | 50.207 | 60.550 | 108.456 | 96.541 | 106.592 | MIMAT0003337 |
|  | hsa-miR-4673 | 49.382 | 59.295 | 73.788 | 56.636 | 75.300 | MIMAT0019755 |
|  | hsa-miR-711 | 49.321 | 61.954 | 67.944 | 50.899 | 65.072 | MIMAT0012734 |
|  | hsa-miR-3202 | 49.294 | 58.700 | 110.453 | 84.664 | 110.464 | MIMAT0015089 |
|  | hsa-miR-4644 | 48.102 | 58.216 | 92.322 | 69.430 | 84.082 | MIMAT0019704 |
|  | hsa-miR-4472 | 43.198 | 40.633 | 38.510 | 31.531 | 34.348 | MIMAT0018999 |
|  | hsa-miR-5096 | 41.952 | 42.411 | 53.809 | 36.708 | 52.571 | MIMAT0020603 |
|  | hsa-miR-6511a-5p | 40.136 | 35.508 | 80.743 | 31.561 | 66.565 | MIMAT0025478 |
|  | hsa-miR-1237-5p | 39.245 | 45.129 | 50.913 | 47.163 | 57.695 | MIMAT0022946 |
|  | hsa-miR-5189 | 37.457 | 40.029 | 46.228 | 35.809 | 44.438 | MIMAT0021120 |
|  | hsa-miR-3713 | 36.284 | 36.453 | 43.413 | 30.710 | 39.913 | MIMAT0018164 |
|  | hsa-miR-4647 | 36.080 | 42.746 | 51.648 | 49.946 | 56.177 | MIMAT0019709 |
|  | hsa-miR-1285-3p | 35.920 | 42.501 | 42.702 | 34.743 | 43.249 | MIMAT0005876 |
|  | hsa-miR-4740-5p | 31.609 | 38.465 | 81.737 | 40.704 | 76.268 | MIMAT0019869 |
|  | hsa-miR-1180 | 31.276 | 39.623 | 33.620 | 32.513 | 32.524 | MIMAT0005825 |
|  | hsa-miR-1229-3p | 25.556 | 53.128 | 54.291 | 55.044 | 63.959 | MIMAT0005584 |
|  | hsa-miR-551b-5p | 18.920 | 28.756 | 25.620 | 27.083 | 26.506 | MIMAT0004794 |
|  | hsa-miR-662 | 15.625 | 62.002 | 90.720 | 75.216 | 86.411 | MIMAT0003325 |
| 10 ≥ >2 | hsa-miR-4299 | 5.827 | 9.122 | 9.576 | 9.108 | 9.472 | MIMAT0016851 |
|  | hsa-miR-422a | 5.103 | 7.636 | 8.118 | 7.485 | 7.908 | MIMAT0001339 |
|  | hsa-miR-197-5p | 4.917 | 7.232 | 7.283 | 7.025 | 6.749 | MIMAT0022691 |
|  | hsa-miR-4488 | 4.539 | 6.223 | 6.411 | 5.753 | 6.369 | MIMAT0019022 |
|  | hsa-miR-4253 | 4.459 | 6.323 | 6.679 | 5.999 | 6.706 | MIMAT0016882 |
|  | hsa-miR-1273c | 4.143 | 5.887 | 5.909 | 4.771 | 5.807 | MIMAT0015017 |
|  | hsa-miR-4672 | 4.092 | 5.752 | 5.953 | 5.519 | 5.829 | MIMAT0019754 |
|  | hsa-miR-550b-2-5p | 4.076 | 5.991 | 6.313 | 5.268 | 5.964 | MIMAT0022737 |
|  | hsa-miR-3934-3p | 4.012 | 4.380 | 4.670 | 3.646 | 4.982 | MIMAT0022975 |
|  | hsa-miR-345-3p | 3.983 | 4.609 | 5.697 | 4.237 | 4.813 | MIMAT0022698 |
|  | hsa-miR-1587 | 3.943 | 5.343 | 6.024 | 5.066 | 5.677 | MIMAT0019077 |
|  | hsa-miR-4788 | 3.865 | 5.404 | 5.498 | 5.266 | 5.129 | MIMAT0019958 |
|  | hsa-miR-4486 | 3.847 | 5.184 | 5.500 | 4.875 | 5.259 | MIMAT0019020 |
|  | hsa-miR-3620-5p | 3.840 | 5.178 | 5.416 | 4.614 | 5.315 | MIMAT0022967 |
|  | hsa-miR-4508 | 3.754 | 4.503 | 5.032 | 4.265 | 4.891 | MIMAT0019045 |
|  | hsa-miR-3156-5p | 3.709 | 5.638 | 5.939 | 5.274 | 5.340 | MIMAT0015030 |
|  | hsa-miR-5088 | 3.682 | 4.084 | 4.989 | 3.840 | 4.763 | MIMAT0021080 |
|  | hsa-miR-1247-3p | 3.587 | 4.647 | 5.058 | 4.225 | 4.926 | MIMAT0022721 |
|  | hsa-miR-4314 | 3.576 | 4.749 | 5.477 | 4.147 | 5.311 | MIMAT0016868 |
|  | hsa-miR-4507 | 3.500 | 4.518 | 5.047 | 4.407 | 4.768 | MIMAT0019044 |
|  | hsa-miR-492 | 3.358 | 3.545 | 4.776 | 3.457 | 4.086 | MIMAT0002812 |
|  | hsa-miR-4665-5p | 3.340 | 3.790 | 4.519 | 3.528 | 4.500 | MIMAT0019739 |
|  | hsa-miR-155-5p | 3.337 | 4.739 | 5.230 | 4.482 | 4.975 | MIMAT0000646 |
|  | hsa-miR-760 | 3.205 | 3.617 | 4.636 | 3.466 | 4.215 | MIMAT0004957 |
|  | hsa-miR-4733-5p | 3.160 | 3.842 | 4.219 | 3.281 | 3.971 | MIMAT0019857 |
|  | hsa-miR-4710 | 3.143 | 3.100 | 3.844 | 2.840 | 3.130 | MIMAT0019815 |
|  | hsa-miR-4505 | 3.024 | 3.937 | 4.534 | 3.794 | 4.270 | MIMAT0019041 |
|  | hsa-miR-4470 | 2.929 | 3.550 | 4.464 | 3.043 | 3.711 | MIMAT0018997 |
|  | hsa-miR-513c-5p | 2.880 | 3.439 | 3.851 | 2.629 | 3.179 | MIMAT0005789 |
|  | hsa-miR-4513 | 2.878 | 3.722 | 4.009 | 3.367 | 3.855 | MIMAT0019050 |
|  | hsa-miR-3609 | 2.850 | 3.691 | 5.583 | 4.544 | 5.758 | MIMAT0017986 |
|  | hsa-miR-514b-5p | 2.756 | 2.889 | 3.620 | 2.480 | 3.002 | MIMAT0015087 |
|  | hsa-miR-664b-5p | 2.714 | 3.322 | 3.971 | 3.078 | 3.725 | MIMAT0022271 |
|  | hsa-miR-4532 | 2.687 | 3.499 | 3.810 | 3.454 | 3.699 | MIMAT0019071 |
|  | hsa-miR-939-5p | 2.686 | 3.053 | 3.690 | 2.864 | 3.411 | MIMAT0004982 |
|  | hsa-miR-623 | 2.683 | 3.276 | 4.473 | 2.271 | 4.498 | MIMAT0003292 |
|  | hsa-miR-5001-5p | 2.663 | 3.570 | 4.093 | 3.515 | 3.839 | MIMAT0021021 |
|  | hsa-miR-4430 | 2.663 | 3.227 | 3.632 | 3.022 | 3.466 | MIMAT0018945 |
|  | hsa-miR-4530 | 2.631 | 3.313 | 3.505 | 3.286 | 3.323 | MIMAT0019069 |
|  | hsa-miR-494 | 2.617 | 2.831 | 3.279 | 2.670 | 3.080 | MIMAT0002816 |
|  | hsa-miR-4697-5p | 2.595 | 3.008 | 3.366 | 2.962 | 3.362 | MIMAT0019791 |
|  | hsa-miR-149-3p | 2.589 | 2.488 | 3.762 | 2.540 | 3.357 | MIMAT0004609 |
|  | hsa-miR-671-5p | 2.587 | 3.233 | 3.745 | 3.061 | 3.455 | MIMAT0003880 |
|  | hsa-miR-4478 | 2.565 | 3.122 | 3.666 | 2.959 | 3.340 | MIMAT0019006 |
|  | hsa-miR-4429 | 2.535 | 2.942 | 3.209 | 2.603 | 3.535 | MIMAT0018944 |
|  | hsa-miR-1268b | 2.505 | 2.791 | 3.408 | 2.642 | 3.117 | MIMAT0018925 |
|  | hsa-miR-6127 | 2.471 | 3.267 | 3.395 | 3.134 | 3.244 | MIMAT0024610 |
|  | hsa-miR-4792 | 2.468 | 2.991 | 3.181 | 2.640 | 2.932 | MIMAT0019964 |
|  | hsa-miR-4446-3p | 2.465 | 2.737 | 3.277 | 2.264 | 2.771 | MIMAT0018965 |
|  | hsa-miR-3679-5p | 2.463 | 3.235 | 3.764 | 2.882 | 3.457 | MIMAT0018104 |
|  | hsa-miR-3137 | 2.458 | 3.535 | 3.772 | 3.377 | 3.565 | MIMAT0015005 |
|  | hsa-miR-4734 | 2.420 | 3.115 | 3.392 | 2.923 | 3.304 | MIMAT0019859 |
|  | hsa-miR-877-5p | 2.407 | 2.461 | 2.875 | 2.328 | 2.699 | MIMAT0004949 |
|  | hsa-miR-4767 | 2.404 | 2.353 | 3.189 | 2.865 | 3.047 | MIMAT0019919 |
|  | hsa-miR-3656 | 2.331 | 2.686 | 3.163 | 2.513 | 2.879 | MIMAT0018076 |
|  | hsa-miR-6075 | 2.329 | 2.993 | 3.376 | 2.795 | 3.098 | MIMAT0023700 |
|  | hsa-miR-4690-5p | 2.319 | 2.658 | 2.934 | 2.459 | 2.740 | MIMAT0019779 |
|  | hsa-miR-4758-5p | 2.315 | 2.604 | 2.780 | 2.154 | 2.578 | MIMAT0019903 |
|  | hsa-miR-3188 | 2.312 | 2.729 | 3.114 | 2.602 | 2.934 | MIMAT0015070 |
|  | hsa-miR-6724-5p | 2.311 | 2.876 | 3.416 | 2.740 | 3.182 | MIMAT0025856 |
|  | hsa-miR-3610 | 2.308 | 2.715 | 3.072 | 2.589 | 2.997 | MIMAT0017987 |
|  | hsa-miR-1469 | 2.298 | 2.619 | 2.962 | 2.296 | 2.842 | MIMAT0007347 |
|  | hsa-miR-3917 | 2.298 | 2.972 | 3.400 | 2.981 | 3.214 | MIMAT0018191 |
|  | hsa-miR-4538 | 2.291 | 2.382 | 3.454 | 2.466 | 2.983 | MIMAT0019081 |
|  | hsa-miR-371b-5p | 2.284 | 2.871 | 3.334 | 2.817 | 3.110 | MIMAT0019892 |
|  | hsa-miR-874 | 2.277 | 2.990 | 3.180 | 2.678 | 2.990 | MIMAT0004911 |
|  | hsa-miR-1224-5p | 2.273 | 2.736 | 3.157 | 2.500 | 3.014 | MIMAT0005458 |
|  | hsa-miR-2392 | 2.249 | 2.507 | 3.172 | 2.352 | 2.941 | MIMAT0019043 |
|  | hsa-miR-4787-5p | 2.222 | 2.790 | 3.153 | 2.711 | 2.942 | MIMAT0019956 |
|  | hsa-miR-4428 | 2.222 | 2.689 | 3.570 | 2.425 | 3.469 | MIMAT0018943 |
|  | hsa-miR-6125 | 2.217 | 2.784 | 3.122 | 2.701 | 2.928 | MIMAT0024598 |
|  | hsa-miR-4322 | 2.204 | 2.626 | 2.909 | 2.323 | 2.680 | MIMAT0016873 |
|  | hsa-miR-1229-5p | 2.196 | 2.533 | 3.073 | 2.359 | 2.869 | MIMAT0022942 |
|  | hsa-miR-548q | 2.196 | 3.000 | 3.028 | 2.634 | 2.907 | MIMAT0011163 |
|  | hsa-miR-4669 | 2.183 | 2.738 | 3.249 | 2.581 | 2.946 | MIMAT0019749 |
|  | hsa-miR-6087 | 2.177 | 2.625 | 2.888 | 2.479 | 2.620 | MIMAT0023712 |
|  | hsa-miR-663b | 2.171 | 2.444 | 3.112 | 2.150 | 2.565 | MIMAT0005867 |
|  | hsa-miR-1225-5p | 2.171 | 2.647 | 2.852 | 2.614 | 2.701 | MIMAT0005572 |
|  | hsa-miR-1185-2-3p | 2.165 | 2.355 | 2.654 | 2.230 | 2.520 | MIMAT0022713 |
|  | hsa-miR-1268a | 2.162 | 2.511 | 2.889 | 2.238 | 2.704 | MIMAT0005922 |
|  | hsa-miR-3607-5p | 2.160 | 2.703 | 2.972 | 2.216 | 2.622 | MIMAT0017984 |
|  | hsa-miR-4783-3p | 2.141 | 2.375 | 2.712 | 2.309 | 2.639 | MIMAT0019947 |
|  | hsa-miR-557 | 2.127 | 2.578 | 2.779 | 2.536 | 2.646 | MIMAT0003221 |
|  | hsa-miR-4270 | 2.127 | 2.403 | 2.778 | 2.311 | 2.547 | MIMAT0016900 |
|  | hsa-miR-4746-3p | 2.121 | 2.572 | 3.063 | 2.692 | 2.987 | MIMAT0019881 |
|  | hsa-miR-575 | 2.099 | 2.556 | 2.604 | 2.362 | 2.548 | MIMAT0003240 |
|  | hsa-miR-652-5p | 2.096 | 2.599 | 2.788 | 2.298 | 2.636 | MIMAT0022709 |
|  | hsa-miR-1185-1-3p | 2.094 | 2.383 | 2.696 | 2.106 | 2.509 | MIMAT0022838 |
|  | hsa-miR-887 | 2.090 | 3.031 | 3.061 | 2.757 | 2.936 | MIMAT0004951 |
|  | hsa-miR-3682-3p | 2.080 | 2.622 | 2.952 | 2.567 | 3.102 | MIMAT0018110 |
|  | hsa-miR-4417 | 2.079 | 2.271 | 2.671 | 2.255 | 2.501 | MIMAT0018929 |
|  | hsa-miR-4695-5p | 2.071 | 2.484 | 2.864 | 2.189 | 2.617 | MIMAT0019788 |
|  | hsa-miR-6722-3p | 2.070 | 2.307 | 2.732 | 2.307 | 2.459 | MIMAT0025854 |
|  | hsa-miR-3652 | 2.069 | 2.472 | 3.036 | 2.377 | 2.890 | MIMAT0018072 |
|  | hsa-miR-1207-5p | 2.058 | 2.465 | 2.777 | 2.384 | 2.618 | MIMAT0005871 |
|  | hsa-miR-6723-5p | 2.057 | 2.170 | 2.449 | 2.110 | 2.342 | MIMAT0025855 |
|  | hsa-miR-513a-5p | 2.056 | 2.417 | 2.712 | 2.175 | 2.635 | MIMAT0002877 |
|  | hsa-miR-937-5p | 2.055 | 2.265 | 2.365 | 2.072 | 2.279 | MIMAT0022938 |
|  | hsa-miR-4634 | 2.054 | 2.258 | 2.522 | 2.196 | 2.348 | MIMAT0019691 |
|  | hsa-miR-125a-3p | 2.052 | 2.561 | 2.794 | 2.408 | 2.622 | MIMAT0004602 |
|  | hsa-miR-4689 | 2.033 | 2.182 | 2.491 | 2.282 | 2.374 | MIMAT0019778 |
|  | hsa-miR-4442 | 2.025 | 2.546 | 2.792 | 2.364 | 2.588 | MIMAT0018960 |
|  | hsa-miR-1227-5p | 2.009 | 2.417 | 2.736 | 2.272 | 2.576 | MIMAT0022941 |
|  | hsa-miR-1226-5p | 2.001 | 2.420 | 2.764 | 2.497 | 2.693 | MIMAT0005576 |

D) MDA-MB-231

| Fold change | microRNA | Relative compression unit | | | | | Accession No. |
| --- | --- | --- | --- | --- | --- | --- | --- |
|  |  | 0.5 | 1 | 2 | 5 | 10 |  |
| > 10 | hsa-miR-3713 | 114.088 | 117.693 | 133.547 | 161.878 | 166.771 | MIMAT0018164 |
|  | hsa-miR-892c-3p | 100.574 | 79.815 | 104.302 | 186.851 | 165.020 | MIMAT0025858 |
|  | hsa-miR-4514 | 98.890 | 86.092 | 115.933 | 134.118 | 110.779 | MIMAT0019051 |
|  | hsa-miR-4749-3p | 98.017 | 76.789 | 99.613 | 92.317 | 100.448 | MIMAT0019886 |
|  | hsa-miR-4486 | 97.084 | 122.303 | 147.911 | 250.556 | 242.823 | MIMAT0019020 |
|  | hsa-miR-4470 | 96.170 | 87.303 | 122.523 | 192.611 | 171.909 | MIMAT0018997 |
|  | hsa-miR-3064-5p | 95.379 | 78.310 | 114.358 | 98.712 | 92.083 | MIMAT0019864 |
|  | hsa-miR-1306-3p | 95.018 | 79.200 | 96.738 | 95.116 | 99.371 | MIMAT0005950 |
|  | hsa-miR-6083 | 95.009 | 81.189 | 112.611 | 136.070 | 121.596 | MIMAT0023708 |
|  | hsa-miR-1238-3p | 93.819 | 75.166 | 114.675 | 101.283 | 104.674 | MIMAT0005593 |
|  | hsa-miR-5587-5p | 93.644 | 78.163 | 93.046 | 98.861 | 99.788 | MIMAT0022289 |
|  | hsa-miR-1225-3p | 92.741 | 87.900 | 90.637 | 111.134 | 130.507 | MIMAT0005573 |
|  | hsa-miR-3925-5p | 91.313 | 76.254 | 101.596 | 136.694 | 126.300 | MIMAT0018200 |
|  | hsa-miR-664a-3p | 87.592 | 71.811 | 91.260 | 123.395 | 140.123 | MIMAT0005949 |
|  | hsa-miR-422a | 85.848 | 87.218 | 117.356 | 129.514 | 107.120 | MIMAT0001339 |
|  | hsa-miR-486-5p | 80.488 | 70.538 | 101.316 | 102.018 | 116.461 | MIMAT0002177 |
|  | hsa-miR-4710 | 80.084 | 75.943 | 94.885 | 122.039 | 106.551 | MIMAT0019815 |
|  | hsa-miR-4297 | 79.969 | 34.154 | 98.645 | 95.196 | 93.386 | MIMAT0016846 |
|  | hsa-miR-509-5p | 78.370 | 76.002 | 102.569 | 146.438 | 125.855 | MIMAT0004779 |
|  | hsa-miR-365b-5p | 76.278 | 74.605 | 99.971 | 118.205 | 113.272 | MIMAT0022833 |
|  | hsa-miR-3692-5p | 75.932 | 68.353 | 106.025 | 169.001 | 152.777 | MIMAT0018121 |
|  | hsa-miR-1247-3p | 75.062 | 66.026 | 101.866 | 111.471 | 102.535 | MIMAT0022721 |
|  | hsa-miR-3180-3p | 71.502 | 62.487 | 40.613 | 96.330 | 85.716 | MIMAT0015058 |
|  | hsa-miR-4648 | 69.999 | 78.815 | 83.275 | 111.790 | 94.722 | MIMAT0019710 |
|  | hsa-miR-3939 | 69.232 | 31.585 | 93.980 | 155.463 | 145.534 | MIMAT0018355 |
|  | hsa-miR-3934-5p | 69.204 | 30.362 | 98.120 | 135.358 | 104.273 | MIMAT0018349 |
|  | hsa-miR-339-3p | 67.329 | 55.871 | 79.290 | 75.223 | 85.965 | MIMAT0004702 |
|  | hsa-miR-4501 | 67.231 | 49.126 | 71.327 | 139.666 | 172.835 | MIMAT0019037 |
|  | hsa-miR-5008-5p | 60.806 | 50.539 | 81.820 | 111.330 | 137.438 | MIMAT0021039 |
|  | hsa-miR-3622b-5p | 57.547 | 91.893 | 108.916 | 137.461 | 117.539 | MIMAT0018005 |
|  | hsa-miR-1207-3p | 50.770 | 46.192 | 67.527 | 76.192 | 91.545 | MIMAT0005872 |
|  | hsa-miR-4485 | 49.282 | 39.517 | 49.743 | 72.730 | 76.824 | MIMAT0019019 |
|  | hsa-miR-1237-3p | 48.342 | 42.125 | 57.943 | 55.216 | 96.475 | MIMAT0005592 |
|  | hsa-miR-1281 | 45.806 | 50.888 | 51.776 | 47.321 | 77.139 | MIMAT0005939 |
|  | hsa-miR-4769-5p | 44.975 | 81.784 | 45.398 | 126.147 | 129.081 | MIMAT0019922 |
|  | hsa-miR-542-5p | 43.500 | 30.575 | 55.816 | 103.924 | 90.944 | MIMAT0003340 |
|  | hsa-miR-4436b-5p | 41.558 | 39.543 | 42.812 | 47.163 | 49.131 | MIMAT0019940 |
|  | hsa-miR-4468 | 41.204 | 33.786 | 47.854 | 116.501 | 107.020 | MIMAT0018995 |
|  | hsa-miR-516b-5p | 40.851 | 40.480 | 45.334 | 75.927 | 68.526 | MIMAT0002859 |
|  | hsa-miR-4446-3p | 40.315 | 36.879 | 63.227 | 169.003 | 175.866 | MIMAT0018965 |
|  | hsa-miR-4472 | 40.076 | 38.046 | 39.893 | 83.858 | 67.713 | MIMAT0018999 |
|  | hsa-miR-3161 | 39.749 | 29.127 | 40.854 | 108.649 | 93.679 | MIMAT0015035 |
|  | hsa-miR-6500-3p | 38.923 | 34.691 | 41.292 | 59.216 | 57.816 | MIMAT0025455 |
|  | hsa-miR-371a-3p | 38.550 | 33.420 | 42.027 | 60.103 | 66.270 | MIMAT0000723 |
|  | hsa-miR-659-3p | 38.343 | 30.508 | 42.686 | 40.788 | 82.364 | MIMAT0003337 |
|  | hsa-miR-191-3p | 37.590 | 48.258 | 20.354 | 44.657 | 98.313 | MIMAT0001618 |
|  | hsa-miR-3689b-3p | 37.404 | 64.835 | 37.885 | 45.722 | 69.445 | MIMAT0018181 |
|  | hsa-miR-378e | 37.152 | 31.991 | 41.782 | 90.898 | 69.381 | MIMAT0018927 |
|  | hsa-miR-1307-5p | 35.041 | 37.962 | 46.161 | 107.115 | 100.155 | MIMAT0022727 |
|  | hsa-miR-4522 | 34.626 | 29.435 | 47.144 | 50.654 | 83.849 | MIMAT0019060 |
|  | hsa-miR-138-2-3p | 32.614 | 18.073 | 17.595 | 107.868 | 97.853 | MIMAT0004596 |
|  | hsa-miR-500a-5p | 22.699 | 22.436 | 28.138 | 68.523 | 85.678 | MIMAT0004773 |
|  | hsa-miR-550a-5p | 15.820 | 15.587 | 18.905 | 27.401 | 23.133 | MIMAT0004800 |
| 10 ≥ >2 | hsa-miR-610 | 3.555 | 3.512 | 4.175 | 4.666 | 4.392 | MIMAT0003278 |
|  | hsa-miR-623 | 3.051 | 3.132 | 3.825 | 4.482 | 4.341 | MIMAT0003292 |
|  | hsa-miR-583 | 2.858 | 2.948 | 2.237 | 5.071 | 4.866 | MIMAT0003248 |
|  | hsa-miR-564 | 2.714 | 2.058 | 3.524 | 3.985 | 3.806 | MIMAT0003228 |

E) CAF1

| Fold change | microRNA | Relative compression unit | | | | | Accession No. |
| --- | --- | --- | --- | --- | --- | --- | --- |
|  |  | 0.5 | 1 | 2 | 5 | 10 |  |
| > 10 | hsa-miR-622 | 172.878 | 151.338 | 164.513 | 177.849 | 178.157 | MIMAT0003291 |
|  | hsa-miR-3138 | 110.037 | 103.617 | 102.368 | 114.698 | 120.634 | MIMAT0015006 |
|  | hsa-miR-4656 | 68.752 | 50.453 | 57.630 | 73.648 | 69.656 | MIMAT0019723 |
|  | hsa-miR-3945 | 3.166 | 2.795 | 3.159 | 3.428 | 3.446 | MIMAT0018361 |
| 10 ≥ >2 | hsa-miR-3605-5p | 2.915 | 2.676 | 2.699 | 2.340 | 2.838 | MIMAT0017981 |
|  | hsa-miR-548q | 2.667 | 2.254 | 2.460 | 2.401 | 2.404 | MIMAT0011163 |
|  | hsa-miR-3137 | 2.643 | 2.362 | 2.527 | 2.643 | 2.712 | MIMAT0015005 |

F) CAF2

| Fold change | microRNA | Relative compression unit | | | | | Accession No. |
| --- | --- | --- | --- | --- | --- | --- | --- |
|  |  | 0.5 | 1 | 2 | 5 | 10 |  |
| > 10 | hsa-miR-3127-5p | 261.923 | 272.762 | 276.670 | 300.927 | 218.217 | MIMAT0014990 |
|  | hsa-miR-1288 | 249.377 | 241.119 | 244.241 | 263.309 | 176.800 | MIMAT0005942 |
|  | hsa-miR-1471 | 239.388 | 242.381 | 237.116 | 272.330 | 258.648 | MIMAT0007349 |
|  | hsa-miR-4665-5p | 231.422 | 303.666 | 286.001 | 278.747 | 60.054 | MIMAT0019739 |
|  | hsa-miR-4455 | 231.402 | 231.405 | 209.577 | 252.355 | 242.529 | MIMAT0018977 |
|  | hsa-miR-4538 | 224.855 | 290.189 | 278.109 | 297.003 | 307.248 | MIMAT0019081 |
|  | hsa-miR-4253 | 221.089 | 223.807 | 232.489 | 251.289 | 153.493 | MIMAT0016882 |
|  | hsa-miR-4743-5p | 220.874 | 215.359 | 219.063 | 233.571 | 191.207 | MIMAT0019874 |
|  | hsa-miR-513b | 217.409 | 312.881 | 293.819 | 314.609 | 272.302 | MIMAT0005788 |
|  | hsa-miR-4690-5p | 211.607 | 225.038 | 214.444 | 243.533 | 215.423 | MIMAT0019779 |
|  | hsa-miR-662 | 204.848 | 217.501 | 209.469 | 205.682 | 147.638 | MIMAT0003325 |
|  | hsa-miR-5190 | 193.629 | 207.372 | 217.472 | 207.835 | 144.966 | MIMAT0021121 |
|  | hsa-miR-4522 | 191.181 | 202.994 | 185.376 | 245.186 | 72.167 | MIMAT0019060 |
|  | hsa-miR-3200-5p | 187.484 | 151.715 | 154.465 | 169.565 | 119.654 | MIMAT0017392 |
|  | hsa-miR-3692-5p | 183.299 | 153.100 | 149.596 | 188.984 | 156.146 | MIMAT0018121 |
|  | hsa-miR-3934-5p | 177.365 | 216.326 | 223.599 | 210.307 | 163.157 | MIMAT0018349 |
|  | hsa-miR-514b-5p | 166.557 | 174.727 | 174.694 | 189.201 | 114.859 | MIMAT0015087 |
|  | hsa-miR-3926 | 159.095 | 163.034 | 161.360 | 173.789 | 146.505 | MIMAT0018201 |
|  | hsa-miR-4755-3p | 156.249 | 199.588 | 177.579 | 195.504 | 147.034 | MIMAT0019896 |
|  | hsa-miR-664a-5p | 146.311 | 67.790 | 67.610 | 76.745 | 71.496 | MIMAT0005948 |
|  | hsa-miR-557 | 139.683 | 133.535 | 142.435 | 153.667 | 127.472 | MIMAT0003221 |
|  | hsa-miR-4685-5p | 134.432 | 153.392 | 135.920 | 157.199 | 74.720 | MIMAT0019771 |
|  | hsa-miR-4769-5p | 133.239 | 157.787 | 133.307 | 135.547 | 78.916 | MIMAT0019922 |
|  | hsa-miR-760 | 132.994 | 148.284 | 148.097 | 157.782 | 29.877 | MIMAT0004957 |
|  | hsa-miR-3154 | 130.806 | 161.188 | 158.130 | 162.276 | 82.253 | MIMAT0015028 |
|  | hsa-miR-149-3p | 115.827 | 103.906 | 110.159 | 116.299 | 43.027 | MIMAT0004609 |
|  | hsa-miR-513c-5p | 105.236 | 167.582 | 163.307 | 180.498 | 85.553 | MIMAT0005789 |
|  | hsa-miR-4513 | 101.886 | 179.880 | 181.730 | 187.375 | 87.813 | MIMAT0019050 |
|  | hsa-miR-4673 | 94.582 | 103.096 | 108.058 | 111.423 | 119.420 | MIMAT0019755 |
|  | hsa-miR-30c-2-3p | 85.894 | 92.447 | 89.912 | 90.102 | 95.915 | MIMAT0004550 |
|  | hsa-miR-550a-3-5p | 84.094 | 91.874 | 85.588 | 89.055 | 75.596 | MIMAT0020925 |
|  | hsa-miR-5008-5p | 79.571 | 80.425 | 143.410 | 151.885 | 73.669 | MIMAT0021039 |
|  | hsa-miR-4647 | 78.779 | 91.123 | 81.463 | 95.213 | 63.264 | MIMAT0019709 |
|  | hsa-miR-4776-5p | 76.122 | 128.424 | 162.167 | 158.971 | 62.533 | MIMAT0019932 |
|  | hsa-miR-5096 | 74.511 | 61.097 | 66.335 | 70.255 | 67.629 | MIMAT0020603 |
|  | hsa-miR-1273e | 70.900 | 113.815 | 66.072 | 113.075 | 81.879 | MIMAT0018079 |
|  | hsa-miR-4688 | 67.776 | 68.766 | 65.803 | 72.194 | 72.296 | MIMAT0019777 |
|  | hsa-miR-345-5p | 67.164 | 64.529 | 67.993 | 68.064 | 69.393 | MIMAT0000772 |
|  | hsa-miR-378a-3p | 64.981 | 143.821 | 132.586 | 126.703 | 62.699 | MIMAT0000732 |
|  | hsa-miR-431-5p | 60.015 | 61.328 | 70.188 | 56.225 | 78.917 | MIMAT0001625 |
|  | hsa-miR-4746-5p | 58.976 | 60.639 | 57.903 | 59.328 | 70.909 | MIMAT0019880 |
|  | hsa-miR-423-3p | 57.528 | 69.031 | 59.741 | 55.011 | 56.553 | MIMAT0001340 |
|  | hsa-miR-22-5p | 55.261 | 68.313 | 61.708 | 49.956 | 63.620 | MIMAT0004495 |
|  | hsa-miR-503-5p | 37.470 | 41.124 | 43.607 | 38.572 | 47.248 | MIMAT0002874 |
|  | hsa-miR-769-3p | 34.378 | 43.348 | 48.380 | 44.070 | 39.391 | MIMAT0003887 |
|  | hsa-miR-4446-3p | 16.217 | 22.063 | 17.917 | 20.396 | 12.483 | MIMAT0018965 |
| 10 ≥ >2 | hsa-miR-494 | 10.514 | 8.804 | 8.537 | 9.730 | 9.438 | MIMAT0002816 |
|  | hsa-miR-4697-5p | 9.393 | 8.771 | 7.844 | 9.176 | 6.740 | MIMAT0019791 |
|  | hsa-miR-513a-5p | 5.547 | 8.864 | 8.079 | 8.304 | 5.877 | MIMAT0002877 |
|  | hsa-miR-99b-3p | 5.316 | 6.929 | 6.548 | 6.488 | 2.415 | MIMAT0004678 |
|  | hsa-miR-4656 | 4.461 | 5.423 | 5.273 | 5.631 | 5.394 | MIMAT0019723 |
|  | hsa-miR-4419a | 4.201 | 4.474 | 4.433 | 4.593 | 4.295 | MIMAT0018931 |
|  | hsa-miR-4738-3p | 3.907 | 4.199 | 4.124 | 4.295 | 3.546 | MIMAT0019867 |
|  | hsa-miR-4478 | 3.711 | 5.053 | 4.516 | 4.775 | 4.781 | MIMAT0019006 |
|  | hsa-miR-1273c | 3.508 | 5.023 | 4.347 | 4.304 | 2.777 | MIMAT0015017 |
|  | hsa-miR-422a | 3.441 | 3.923 | 3.773 | 3.544 | 2.200 | MIMAT0001339 |
|  | hsa-miR-1587 | 3.436 | 3.562 | 3.300 | 4.099 | 3.736 | MIMAT0019077 |
|  | hsa-miR-4515 | 3.436 | 3.255 | 3.022 | 3.421 | 2.803 | MIMAT0019052 |
|  | hsa-miR-424-3p | 3.421 | 3.511 | 3.163 | 3.557 | 4.002 | MIMAT0004749 |
|  | hsa-miR-1972 | 3.411 | 2.602 | 2.816 | 3.007 | 2.246 | MIMAT0009447 |
|  | hsa-miR-370 | 3.348 | 3.444 | 3.435 | 3.630 | 3.007 | MIMAT0000722 |
|  | hsa-miR-6132 | 3.245 | 3.623 | 3.368 | 3.523 | 3.420 | MIMAT0024616 |
|  | hsa-miR-550b-2-5p | 3.183 | 3.489 | 3.324 | 3.309 | 2.992 | MIMAT0022737 |
|  | hsa-miR-6129 | 3.169 | 3.307 | 3.172 | 3.203 | 2.862 | MIMAT0024613 |
|  | hsa-miR-1469 | 3.015 | 3.652 | 3.455 | 3.762 | 2.541 | MIMAT0007347 |
|  | hsa-miR-1273f | 3.007 | 2.682 | 2.702 | 2.912 | 3.146 | MIMAT0020601 |
|  | hsa-miR-575 | 3.006 | 3.221 | 3.047 | 3.078 | 3.051 | MIMAT0003240 |
|  | hsa-miR-198 | 2.976 | 3.067 | 2.916 | 2.887 | 2.821 | MIMAT0000228 |
|  | hsa-miR-1275 | 2.968 | 2.495 | 2.431 | 2.769 | 2.773 | MIMAT0005929 |
|  | hsa-miR-3125 | 2.934 | 2.988 | 2.991 | 3.049 | 2.387 | MIMAT0014988 |
|  | hsa-miR-3202 | 2.919 | 3.397 | 3.255 | 3.334 | 3.184 | MIMAT0015089 |
|  | hsa-miR-4728-5p | 2.918 | 3.348 | 3.207 | 3.234 | 3.441 | MIMAT0019849 |
|  | hsa-miR-574-5p | 2.850 | 2.686 | 2.600 | 2.995 | 3.038 | MIMAT0004795 |
|  | hsa-miR-5194 | 2.850 | 2.921 | 3.178 | 3.244 | 2.744 | MIMAT0021125 |
|  | hsa-miR-3198 | 2.803 | 3.103 | 2.986 | 3.022 | 2.465 | MIMAT0015083 |
|  | hsa-miR-4758-5p | 2.745 | 3.111 | 2.927 | 3.035 | 2.351 | MIMAT0019903 |
|  | hsa-miR-6717-5p | 2.722 | 3.199 | 3.015 | 3.169 | 2.524 | MIMAT0025846 |
|  | hsa-miR-4428 | 2.692 | 3.808 | 3.638 | 3.794 | 4.166 | MIMAT0018943 |
|  | hsa-miR-1305 | 2.677 | 2.867 | 2.798 | 2.880 | 2.261 | MIMAT0005893 |
|  | hsa-miR-4713-3p | 2.671 | 2.684 | 2.728 | 2.771 | 2.292 | MIMAT0019821 |
|  | hsa-miR-664b-5p | 2.644 | 2.071 | 2.159 | 2.158 | 2.202 | MIMAT0022271 |
|  | hsa-miR-3676-5p | 2.632 | 2.600 | 2.549 | 2.139 | 2.810 | MIMAT0022734 |
|  | hsa-miR-4741 | 2.605 | 2.674 | 2.556 | 2.691 | 2.457 | MIMAT0019871 |
|  | hsa-miR-5581-5p | 2.604 | 2.606 | 2.687 | 2.686 | 2.204 | MIMAT0022275 |
|  | hsa-miR-6131 | 2.560 | 2.691 | 2.710 | 2.776 | 2.389 | MIMAT0024615 |
|  | hsa-miR-4481 | 2.537 | 3.085 | 3.039 | 3.112 | 2.753 | MIMAT0019015 |
|  | hsa-miR-30c-1-3p | 2.497 | 2.377 | 2.296 | 2.603 | 2.100 | MIMAT0004674 |
|  | hsa-miR-3652 | 2.497 | 2.538 | 2.390 | 2.867 | 2.497 | MIMAT0018072 |
|  | hsa-miR-671-5p | 2.472 | 2.503 | 2.454 | 2.501 | 2.578 | MIMAT0003880 |
|  | hsa-miR-3194-5p | 2.457 | 2.468 | 2.416 | 2.675 | 2.182 | MIMAT0015078 |
|  | hsa-miR-1227-5p | 2.437 | 2.469 | 2.399 | 2.676 | 2.632 | MIMAT0022941 |
|  | hsa-miR-6068 | 2.421 | 2.432 | 2.310 | 2.542 | 2.573 | MIMAT0023693 |
|  | hsa-miR-4716-3p | 2.392 | 2.533 | 2.504 | 2.563 | 2.080 | MIMAT0019827 |
|  | hsa-miR-6124 | 2.367 | 2.717 | 2.515 | 2.779 | 2.109 | MIMAT0024597 |
|  | hsa-miR-1914-3p | 2.366 | 2.421 | 2.456 | 2.517 | 2.250 | MIMAT0007890 |
|  | hsa-miR-572 | 2.362 | 2.129 | 2.230 | 2.537 | 2.486 | MIMAT0003237 |
|  | hsa-miR-3137 | 2.350 | 2.300 | 2.376 | 2.457 | 2.023 | MIMAT0015005 |
|  | hsa-miR-4646-5p | 2.336 | 2.443 | 2.454 | 2.419 | 2.487 | MIMAT0019707 |
|  | hsa-miR-6075 | 2.304 | 2.614 | 2.415 | 2.660 | 2.401 | MIMAT0023700 |
|  | hsa-miR-3135b | 2.301 | 2.093 | 2.120 | 2.335 | 2.348 | MIMAT0018985 |
|  | hsa-miR-6127 | 2.286 | 2.315 | 2.331 | 2.389 | 2.333 | MIMAT0024610 |
|  | hsa-miR-135a-3p | 2.254 | 2.246 | 2.184 | 2.470 | 2.165 | MIMAT0004595 |
|  | hsa-miR-1249 | 2.220 | 2.192 | 2.239 | 2.310 | 3.057 | MIMAT0005901 |
|  | hsa-miR-4534 | 2.219 | 2.139 | 2.077 | 2.236 | 2.226 | MIMAT0019073 |
|  | hsa-miR-4298 | 2.188 | 2.058 | 2.044 | 2.141 | 2.103 | MIMAT0016852 |
|  | hsa-miR-345-3p | 2.178 | 2.200 | 2.066 | 2.219 | 2.109 | MIMAT0022698 |
|  | hsa-miR-4530 | 2.173 | 2.180 | 2.088 | 2.162 | 2.218 | MIMAT0019069 |
|  | hsa-miR-663a | 2.167 | 2.031 | 2.052 | 2.148 | 2.100 | MIMAT0003326 |
|  | hsa-miR-4299 | 2.148 | 2.497 | 2.515 | 2.389 | 2.225 | MIMAT0016851 |
|  | hsa-miR-5006-5p | 2.093 | 2.158 | 2.057 | 2.135 | 2.346 | MIMAT0021033 |
|  | hsa-miR-4667-5p | 2.080 | 2.333 | 2.259 | 2.135 | 2.100 | MIMAT0019743 |
|  | hsa-miR-4505 | 2.052 | 2.173 | 2.016 | 2.301 | 2.098 | MIMAT0019041 |
|  | hsa-miR-34b-5p | 2.051 | 2.464 | 2.345 | 2.232 | 3.083 | MIMAT0000685 |

G) CAF3

| Fold change | microRNA | Relative compression unit | | | | | Accession No. |
| --- | --- | --- | --- | --- | --- | --- | --- |
|  |  | 0.5 | 1 | 2 | 5 | 10 |  |
| > 10 | hsa-miR-1288 | 134.330 | 140.579 | 246.698 | 168.504 | 276.526 | MIMAT0005942 |
|  | hsa-miR-3158-3p | 122.245 | 131.690 | 154.285 | 124.698 | 152.178 | MIMAT0015032 |
|  | hsa-miR-450a-5p | 78.433 | 68.545 | 177.497 | 163.150 | 278.808 | MIMAT0001545 |
|  | hsa-miR-301a-3p | 71.569 | 69.693 | 97.020 | 65.313 | 162.331 | MIMAT0000688 |
|  | hsa-miR-4446-3p | 69.794 | 170.408 | 61.356 | 185.844 | 70.264 | MIMAT0018965 |
|  | hsa-miR-378i | 68.412 | 81.881 | 109.213 | 69.937 | 122.826 | MIMAT0019074 |
|  | hsa-miR-1271-5p | 66.668 | 72.197 | 106.282 | 61.109 | 116.641 | MIMAT0005796 |
|  | hsa-miR-516a-5p | 66.597 | 66.056 | 95.611 | 140.622 | 223.489 | MIMAT0004770 |
|  | hsa-miR-4769-5p | 64.509 | 114.812 | 72.792 | 157.791 | 95.687 | MIMAT0019922 |
|  | hsa-miR-519e-5p | 63.432 | 62.059 | 76.013 | 90.886 | 121.208 | MIMAT0002828 |
|  | hsa-miR-431-5p | 63.300 | 79.378 | 97.504 | 71.386 | 109.119 | MIMAT0001625 |
|  | hsa-miR-455-5p | 62.005 | 72.838 | 100.072 | 68.815 | 105.430 | MIMAT0003150 |
|  | hsa-miR-126-3p | 59.115 | 59.956 | 100.886 | 65.950 | 112.525 | MIMAT0000445 |
|  | hsa-miR-345-3p | 58.181 | 138.608 | 148.859 | 168.406 | 160.066 | MIMAT0022698 |
|  | hsa-miR-758-3p | 57.558 | 57.828 | 97.263 | 33.185 | 142.553 | MIMAT0003879 |
|  | hsa-miR-214-5p | 56.003 | 68.383 | 87.164 | 73.596 | 100.365 | MIMAT0004564 |
|  | hsa-miR-550a-3-5p | 55.691 | 65.749 | 74.594 | 84.537 | 71.694 | MIMAT0020925 |
|  | hsa-miR-181a-3p | 55.174 | 58.483 | 79.720 | 59.656 | 149.088 | MIMAT0000270 |
|  | hsa-miR-4743-5p | 54.641 | 65.802 | 66.937 | 154.416 | 172.176 | MIMAT0019874 |
|  | hsa-miR-590-5p | 53.023 | 125.763 | 162.778 | 127.995 | 177.108 | MIMAT0003258 |
|  | hsa-miR-623 | 38.586 | 37.686 | 41.755 | 51.199 | 55.051 | MIMAT0003292 |
| 10 ≥ >2 | hsa-miR-422a | 4.070 | 4.566 | 4.970 | 5.720 | 4.156 | MIMAT0001339 |
|  | hsa-miR-3162-3p | 2.604 | 3.574 | 6.917 | 2.918 | 12.248 | MIMAT0019213 |
|  | hsa-miR-520b | 2.269 | 2.809 | 3.410 | 2.945 | 3.053 | MIMAT0002843 |
|  | hsa-miR-218-5p | 2.207 | 2.337 | 3.584 | 3.018 | 4.542 | MIMAT0000275 |
|  | hsa-miR-4755-3p | 2.103 | 2.146 | 2.991 | 3.530 | 3.809 | MIMAT0019896 |
|  | hsa-miR-337-3p | 2.076 | 2.139 | 3.083 | 2.721 | 4.834 | MIMAT0000754 |
|  | hsa-miR-503-5p | 2.038 | 2.138 | 2.515 | 2.214 | 2.486 | MIMAT0002874 |
|  | hsa-miR-539-5p | 2.030 | 2.311 | 2.921 | 2.128 | 3.522 | MIMAT0003163 |

H) CAF4

| Fold change | microRNA | Relative compression unit | | | | | Accession No. |
| --- | --- | --- | --- | --- | --- | --- | --- |
|  |  | 0.5 | 1 | 2 | 5 | 10 |  |
| > 10 | hsa-miR-3138 | 213.135 | 229.133 | 213.931 | 266.973 | 140.466 | MIMAT0015006 |
|  | hsa-miR-3127-5p | 197.668 | 193.740 | 190.997 | 203.828 | 121.103 | MIMAT0014990 |
|  | hsa-miR-5190 | 188.890 | 211.566 | 176.857 | 224.929 | 62.427 | MIMAT0021121 |
|  | hsa-miR-3194-5p | 147.474 | 167.051 | 156.887 | 183.832 | 109.520 | MIMAT0015078 |
|  | hsa-miR-4472 | 78.068 | 71.546 | 86.706 | 97.964 | 73.286 | MIMAT0018999 |
|  | hsa-miR-4656 | 77.179 | 95.762 | 153.156 | 104.113 | 78.988 | MIMAT0019723 |
|  | hsa-miR-4455 | 76.774 | 74.002 | 156.375 | 158.662 | 130.657 | MIMAT0018977 |
|  | hsa-miR-1469 | 76.750 | 76.633 | 85.259 | 106.709 | 61.494 | MIMAT0007347 |
|  | hsa-miR-4750-5p | 74.790 | 81.092 | 106.418 | 119.822 | 87.669 | MIMAT0019887 |
|  | hsa-miR-4769-5p | 70.966 | 89.728 | 128.885 | 181.724 | 64.621 | MIMAT0019922 |
|  | hsa-miR-198 | 69.275 | 69.990 | 62.287 | 87.890 | 56.224 | MIMAT0000228 |
|  | hsa-miR-3154 | 67.846 | 156.877 | 145.536 | 176.491 | 59.928 | MIMAT0015028 |
|  | hsa-miR-514b-5p | 66.632 | 76.787 | 69.805 | 93.968 | 66.323 | MIMAT0015087 |
|  | hsa-miR-4446-3p | 62.857 | 80.128 | 79.955 | 120.282 | 64.326 | MIMAT0018965 |
|  | hsa-miR-345-5p | 39.123 | 46.365 | 50.320 | 106.619 | 56.587 | MIMAT0000772 |
|  | hsa-miR-339-3p | 35.323 | 74.828 | 55.748 | 62.730 | 25.421 | MIMAT0004702 |
| 10 ≥ >2 | hsa-miR-513b | 3.423 | 5.480 | 4.541 | 5.374 | 2.219 | MIMAT0005788 |
