# Supplementary material for: Transcriptome-wide analysis of compression-induced microRNA expression alteration in breast cancer for mining therapeutic targets
Source: Oncotarget. 2016 Mar 24;7(19):27468–78. doi: 10.18632/oncotarget.8322 (PMC5053664; doi:10.18632/oncotarget.8322)
Supplement: Supplementary file 3 [file oncotarget-07-27468-s003.docx]

**Supplementary Table S2. Putative target genes of compression-upregulated microRNAs.**

| Cell | microRNA | Putative target genes |
| --- | --- | --- |
| BT-474 | hsa-miR-671-5p | FAM110B XKR5 |
|  | hsa-miR-4486 | INMT EMILIN2 |
|  | hsa-miR-664b-5p |  |
|  | hsa-miR-484 |  |
|  | hsa-miR-664a-3p | RFX3 TCEAL7 C1QTNF7 RPP30 LRRK2 DMRTA1 |
|  | hsa-miR-4656 | TSPAN7 HIVEP3 PKIA ZSWIM3 TMEM201 |
|  | hsa-miR-340-5p | KAT6B ZNF503 DYNC1I1 PHTF2 GALNT13 NCAM2 FSD1L ANO2 |
|  | hsa-miR-1249-3p | GRB14 |
|  | hsa-miR-7-1-3p | FSD1L FGL2 MAT1A FRMD3 CALCR FZD8 SLC12A5 PLEKHG4 PTPRC TRHDE ARL13B TRDN PCDH11Y ROBO2 HERC2 RNF180 DLG2 FBXL17 LINGO2 PMP2 GALNT13 |
|  | hsa-miR-1281 | SLC12A5 |
|  | hsa-miR-4299 | DIO2 NCAM1 |
|  | hsa-miR-197-5p | AKR1B1 ZNF239 CCDC129 MTUS2 PHC1 |
| MCF7 | hsa-miR-4733-5p | ZDHHC21 TARDBP PAN3 |
|  | hsa-miR-617 |  |
|  | hsa-miR-557 | TET1 BEND4 |
|  | hsa-miR-4508 | TBX1 |
|  | hsa-miR-663b | DCX PLEKHA8 |
|  | hsa-miR-5191 |  |
|  | hsa-miR-601 | B3GNT9 |
|  | hsa-miR-423-3p |  |
| SK-BR-3 | hsa-miR-617 |  |
|  | hsa-miR-664a-5p | SIM2 TIMP3 |
|  | hsa-miR-99b-3p |  |
|  | hsa-miR-628-3p | SLAIN2 |
|  | hsa-miR-3654 | ARHGAP20 |
|  | hsa-miR-4282 | RUNDC3B KCNK2 MYO5C ZC3H12C DPY19L3 PROS1 FAR1 ALDH5A1 HCN1 ABCB5 OGN TRIM36 NEK1 KCNC1 F5 ONECUT2 PLAA |
|  | hsa-miR-4539 | RIMS2 |
|  | hsa-miR-202-3p | PPARGC1B TECPR2 LBH AGBL2 EYA3 LIN52 LAMA1 |
|  | hsa-miR-3605-5p | KIAA0408 |
|  | hsa-miR-708-5p | HOXA1 ZNF569 |
|  | hsa-miR-610 | CYBRD1 |
|  | hsa-miR-125b-1-3p |  |
|  | hsa-miR-3689f | PIK3CG ARSJ HOXB9 HSF5 WLS |
|  | hsa-miR-10b-3p | KRTAP4-3 VASH2 ZNF140 |
|  | hsa-miR-3622b-5p | RNFT2 MSTN |
|  | hsa-miR-3180-3p | SCRT1 |
|  | hsa-miR-542-5p |  |
|  | hsa-miR-1307-5p |  |
|  | hsa-miR-4514 | KIF3C ERCC4 GABRB3 CD5L PCSK7 LBH ANGPT2 DHDDS MAP1A RIMS4 |
|  | hsa-miR-4776-5p | PAFAH1B2 RIMS2 TEX15 |
|  | hsa-miR-4664-3p |  |
|  | hsa-miR-498 | LEP |
|  | hsa-miR-6511b-5p | TMEM98 TWIST1 FNDC5 ZNF132 |
|  | hsa-miR-659-3p | SERPINB3 |
|  | hsa-miR-4673 | SHC2 CAMTA1 TRIM36 ZNF132 SLC34A1 |
|  | hsa-miR-711 |  |
|  | hsa-miR-3202 | JPH4 EYA3 PPHLN1 PPARGC1B SIRPA RIMS4 CAPZB FAM155B OSBP2 SLC26A9 DLG2 GLYCTK PFKFB2 |
|  | hsa-miR-4644 | FIGNL2 EYA3 KIF3C CAPZB ADH4 CNTLN CAMK1G LPAR5 DLG2 CDH1 SIX3 LRIT1 DTX3 SDC3 ELMOD3 CYP4V2 |
|  | hsa-miR-4472 | DTX3 ADORA3 SIRPA HIP1 C6orf141 GIMAP4 DAGLA |
|  | hsa-miR-5096 | RIMS2 |
|  | hsa-miR-6511a-5p | JPH3 MPL GRIK3 |
|  | hsa-miR-1237-5p |  |
|  | hsa-miR-5189 | CMKLR1 KCNK2 NFAM1 PPP2R2B GPR26 |
|  | hsa-miR-3713 |  |
|  | hsa-miR-4647 | COL1A2 FAR1 |
|  | hsa-miR-1285-3p | CMKLR1 KCNK2 NFAM1 GPR26 PAFAH1B2 PPP2R2B GRM6 DCDC5 TUB |
|  | hsa-miR-4740-5p | JPH3 |
|  | hsa-miR-1180 |  |
|  | hsa-miR-1229-3p |  |
|  | hsa-miR-551b-5p | SLC35F1 ZC3H12C AGTR1 SLAIN2 PPHLN1 FAR1 TAT RIMS2 CUL3 DLG2 GABRB3 PFKFB2 BMPER KIAA0408 |
|  | hsa-miR-662 |  |
| MDA-MB-231 | hsa-miR-3713 |  |
|  | hsa-miR-892c-3p | PHF6 BZW1 CTTNBP2 RAB11FIP2 LRRC8B TNPO1 TMEM170B RAB11A LOC389831 PRPF4B |
|  | hsa-miR-4514 | FBXL20 GCC1 MSI2 UTRN NAV2 TRAF3 ERCC4 PCSK7 EIF4G3 WSCD2 PCDHB10 MAP3K13 OR2C3 SSR1 |
|  | hsa-miR-4749-3p | FURIN STAG2 OAZ2 |
|  | hsa-miR-4486 | APOBEC3D PKD1 |
|  | hsa-miR-4470 | SORBS2 FAM174B ALCAM SNX4 SCN7A |
|  | hsa-miR-3064-5p | PIP4K2B TRAF3 CA10 ANKRD36 FAM178A GOLGA7B IL1RL1 ZNRF2 PADI2 |
|  | hsa-miR-1306-3p |  |
|  | hsa-miR-6083 |  |
|  | hsa-miR-1238-3p |  |
|  | hsa-miR-5587-5p | NSF PHF6 RAB14 |
|  | hsa-miR-1225-3p | OAZ2 TRAM1 |
|  | hsa-miR-3925-5p | PRICKLE2 TRAM1 COL19A1 JAZF1 GDNF CBX5 PRKCE SRSF6 MED13L FLNA CELF3 MMRN1 KANK1 |
|  | hsa-miR-664a-3p | ABCB10 LRRC8B SLC5A7 DENND4C ANKRD28 BCL2A1 FAR1 RPS6KB1 DNM3 BCAR3 SLC38A2 SORBS2 C5orf24 |
|  | hsa-miR-422a | FLT1 SHE EIF4G3 FAM178A |
|  | hsa-miR-486-5p | ZNF331 PLAGL2 NCKAP5 TRIM36 CD247 |
|  | hsa-miR-4710 | JAZF1 BHMT2 |
|  | hsa-miR-4297 | ANO3 KDM5B MFHAS1 TBL2 FAF2 POPDC2 SPATA2L KIF1B UNC5D PPFIA1 C6orf89 |
|  | hsa-miR-509-5p | ERLIN2 TARDBP CAMTA1 RAB11A PDE11A DNM3 GMFB MED13L TNPO1 PIP4K2B SHROOM1 |
|  | hsa-miR-365b-5p |  |
|  | hsa-miR-3692-5p | ARL3 SEC14L4 ZMYND8 JAZF1 SV2A BCL2L13 PADI2 |
|  | hsa-miR-1247-3p |  |
|  | hsa-miR-3180-3p | WSCD2 |
|  | hsa-miR-4648 | CACNA1C LPP TRPM3 ARHGAP32 CTSE |
|  | hsa-miR-3939 |  |
|  | hsa-miR-3934-5p | TXNRD2 FARP1 ALCAM SORBS2 ZNF614 |
|  | hsa-miR-339-3p |  |
|  | hsa-miR-4501 | CA10 KIAA0040 HCN1 |
|  | hsa-miR-5008-5p | TBL2 BSND EPB41L1 |
|  | hsa-miR-3622b-5p | STAG2 GTF2I PLCB4 CBX5 IMPA1 |
|  | hsa-miR-1207-3p | CSMD3 ATP2B4 RAB11FIP2 RPS6KB1 EPB41L1 SPPL2B |
|  | hsa-miR-4485 |  |
|  | hsa-miR-1237-3p | SYAP1 HAUS2 HIP1 FRMD6 KANSL3 ERLIN2 |
|  | hsa-miR-1281 |  |
|  | hsa-miR-4769-5p | WSCD2 PIP4K2B MMP25 RAG2 |
|  | hsa-miR-542-5p | SGCZ HDDC2 |
|  | hsa-miR-4436b-5p | STAG2 TMEM170B CTTNBP2 PRNP PHEX HSPA8 TRIM36 |
|  | hsa-miR-4468 | CDC42SE2 DCLK1 FBXL20 EFNA3 |
|  | hsa-miR-516b-5p | LDOC1L NSF B4GALT1 AGPAT3 |
|  | hsa-miR-4446-3p | SSR1 CYBRD1 CELF3 C1orf162 ATP6V0D1 EPHA10 ZMAT4 |
|  | hsa-miR-4472 | GDI1 NTSR1 DLX3 TIE1 ADORA3 CYB5R3 NAV2 HRK EPB41L1 CACNA1C ZBTB4 TSPAN9 CNDP1 HIP1 C5orf24 FAM169A |
|  | hsa-miR-3161 | FNDC3A PDZD7 KPNA4 |
|  | hsa-miR-6500-3p |  |
|  | hsa-miR-371a-3p |  |
|  | hsa-miR-659-3p | FAM107B LRIG3 SCML2 FRMD6 PSMF1 |
|  | hsa-miR-191-3p |  |
|  | hsa-miR-3689b-3p | RPH3AL ANKRD40 GDI1 ELK1 SLC36A3 SLC8A1 GALNT2 RAB5B CBX5 ERLIN2 PLAGL2 NTRK3 FURIN RBM8A LHPP |
|  | hsa-miR-378e | FLT1 FAM178A EIF4G3 |
|  | hsa-miR-1307-5p |  |
|  | hsa-miR-4522 | HLA-DPB1 APOBEC3B YPEL5 |
|  | hsa-miR-138-2-3p | ANKRD28 CAMTA1 UBE2V2 C5orf24 IMPA1 TLL1 |
|  | hsa-miR-500a-5p | SUB1 SERBP1 ZMAT4 SKA3 CLIC4 RPS6KB1 PIGN |
|  | hsa-miR-550a-5p | RTN4RL1 MTUS2 DENND4C |
| CAF1 | hsa-miR-622 | PHF20L1 |
|  | hsa-miR-3138 |  |
|  | hsa-miR-4656 | MOBP LPHN3 FA2H |
| CAF2 | hsa-miR-3127-5p | ANKRD34A NCBP1 |
|  | hsa-miR-1288 |  |
|  | hsa-miR-1471 |  |
|  | hsa-miR-4665-5p | PCDHGA8 NAALADL2 FAM50B MAPK9 |
|  | hsa-miR-4455 | ANKRD34A SLC48A1 HUS1 XKRX ANKRD39 GPAM DCDC5 KCNAB2 |
|  | hsa-miR-4538 | SUB1 |
|  | hsa-miR-4253 | PPT1 |
|  | hsa-miR-4743-5p |  |
|  | hsa-miR-513b | SEL1L IMPACT GCH1 KIAA1211 DAZAP1 TRAPPC2 |
|  | hsa-miR-4690-5p | SEL1L SKA2 |
|  | hsa-miR-662 |  |
|  | hsa-miR-5190 |  |
|  | hsa-miR-4522 | CNR1 LINGO1 |
|  | hsa-miR-3200-5p | LANCL1 |
|  | hsa-miR-3692-5p | GPM6B NEUROD1 DOK7 MYO1D |
|  | hsa-miR-3934-5p | SLC43A2 |
|  | hsa-miR-514b-5p | YTHDC2 IPCEF1 TM7SF3 |
|  | hsa-miR-3926 | RTN4IP1 LOXL1 TMEM50B |
|  | hsa-miR-4755-3p | PCDH9 SLC43A2 PIAS4 CEP41 STOML1 EFCAB5 IFNAR1 ANKRD34A SLC48A1 |
|  | hsa-miR-664a-5p |  |
|  | hsa-miR-557 | PCDH9 TMEM56 NELL2 PNN IFFO1 EMILIN2 |
|  | hsa-miR-4685-5p | SLC43A2 GAS7 LRRC20 PBX1 ANKRD34A FAM78A |
|  | hsa-miR-4769-5p |  |
|  | hsa-miR-760 | TOMM40L KCNJ5 SEL1L SLC43A2 SLCO3A1 TRIM14 |
|  | hsa-miR-3154 | SLC35B4 SLC25A15 SEL1L FHL1 ADD2 B9D1 ALDH1A2 GJA5 FAM78A RMND5B |
|  | hsa-miR-149-3p | DOLPP1 HOXC4 CCDC64 TRIM14 TMEM110 TMEM201 DNAJC30 PBX1 CD300LG CYHR1 PAFAH2 |
|  | hsa-miR-513c-5p | YTHDC2 IPCEF1 TM7SF3 |
|  | hsa-miR-4513 | ZNF594 |
|  | hsa-miR-4673 | SLC35B4 TMEM126B ZNF608 POLR3H GAS7 |
|  | hsa-miR-30c-2-3p | UST NAMPT FABP4 ADD2 |
|  | hsa-miR-550a-3-5p | DERA |
|  | hsa-miR-5008-5p | LINGO1 RTP2 |
|  | hsa-miR-4647 |  |
|  | hsa-miR-4776-5p |  |
|  | hsa-miR-5096 | FAM120C KCNJ12 GATA4 STARD13 |
|  | hsa-miR-1273e | TRAPPC2 APITD1 |
|  | hsa-miR-4688 | PCDH9 TSPAN2 SEL1L |
|  | hsa-miR-345-5p |  |
|  | hsa-miR-378a-3p | ELAC1 |
|  | hsa-miR-431-5p | KCNJ2 |
|  | hsa-miR-4746-5p |  |
|  | hsa-miR-423-3p |  |
|  | hsa-miR-22-5p | TLE4 CNR1 MMAA |
|  | hsa-miR-503-5p |  |
|  | hsa-miR-769-3p | FAM19A2 TCHP |
|  | hsa-miR-4446-3p | PAIP1 SLC35B4 GAS7 IVD |
| CAF3 | hsa-miR-1288 |  |
|  | hsa-miR-3158-3p | NEDD4L |
|  | hsa-miR-450a-5p |  |
|  | hsa-miR-301a-3p | ARAP2 SYT6 KIAA1211 ALDH3A2 SLC12A7 DLL1 |
|  | hsa-miR-4446-3p | PAIP1 GAS7 LONRF2 |
|  | hsa-miR-378i | ELAC1 |
|  | hsa-miR-1271-5p | TMEM170B SLC16A9 SLC39A1 CHIC1 XKR4 MFAP3L |
|  | hsa-miR-516a-5p |  |
|  | hsa-miR-4769-5p | MEF2C ACAN SPRR4 |
|  | hsa-miR-519e-5p | ETV1 TSEN2 |
|  | hsa-miR-431-5p | KCNJ2 |
|  | hsa-miR-455-5p |  |
|  | hsa-miR-126-3p |  |
|  | hsa-miR-345-3p | SLC24A2 ABCC5 FRAT1 NEDD9 |
|  | hsa-miR-758-3p | UNC5D RBM15B |
|  | hsa-miR-214-5p | CEP68 |
|  | hsa-miR-550a-3-5p | MTUS2 |
|  | hsa-miR-181a-3p |  |
|  | hsa-miR-4743-5p |  |
|  | hsa-miR-590-5p |  |
|  | hsa-miR-623 | MEF2C |
| CAF4 | hsa-miR-3138 | COL19A1 FPGT KCTD1 |
|  | hsa-miR-3127-5p | C15orf41 |
|  | hsa-miR-5190 | SDAD1 CXCL9 |
|  | hsa-miR-3194-5p |  |
|  | hsa-miR-4472 | NTSR1 DLX3 TRANK1 SEMA4G KCNJ4 SERP2 KIAA1549 GRIP2 PDLIM3 |
|  | hsa-miR-4656 | SDAD1 TMEM201 |
|  | hsa-miR-4455 | ATP8A1 MYL1 SLC48A1 SCN2B ZBTB24 POLR3B GPAM SLITRK5 PTPN4 RAB3D |
|  | hsa-miR-1469 |  |
|  | hsa-miR-4750-5p |  |
|  | hsa-miR-4769-5p |  |
|  | hsa-miR-198 | PDCD1LG2 SLITRK5 PBX1 SPATA6 |
|  | hsa-miR-3154 | SLC35B4 SLC1A3 RNGTT KIAA1549 SYT1 KIAA1407 KY B9D1 |
|  | hsa-miR-514b-5p | IPCEF1 BCL11B |
|  | hsa-miR-4446-3p | ABCG4 PAIP1 SLC35B4 CELF3 LONRF2 FAM43B ANKRD1 ORC5 KIF24 |
|  | hsa-miR-345-5p | KIAA1549 |
|  | hsa-miR-339-3p |  |
